# Supplementary material for: Long-term tolerance of islet allografts in nonhuman primates induced by apoptotic donor leukocytes
Source: Nat Commun. 2019 Aug 2;10:3495. doi: 10.1038/s41467-019-11338-y (PMC6677762; doi:10.1038/s41467-019-11338-y)
Supplement: Supplementary file 1 — Supplementary Information [file 41467_2019_11338_MOESM1_ESM.pdf]

Long-term tolerance of islet allografts in nonhuman primates induced by apoptotic donor leukocytes

Singh et al.

Supplementary Information

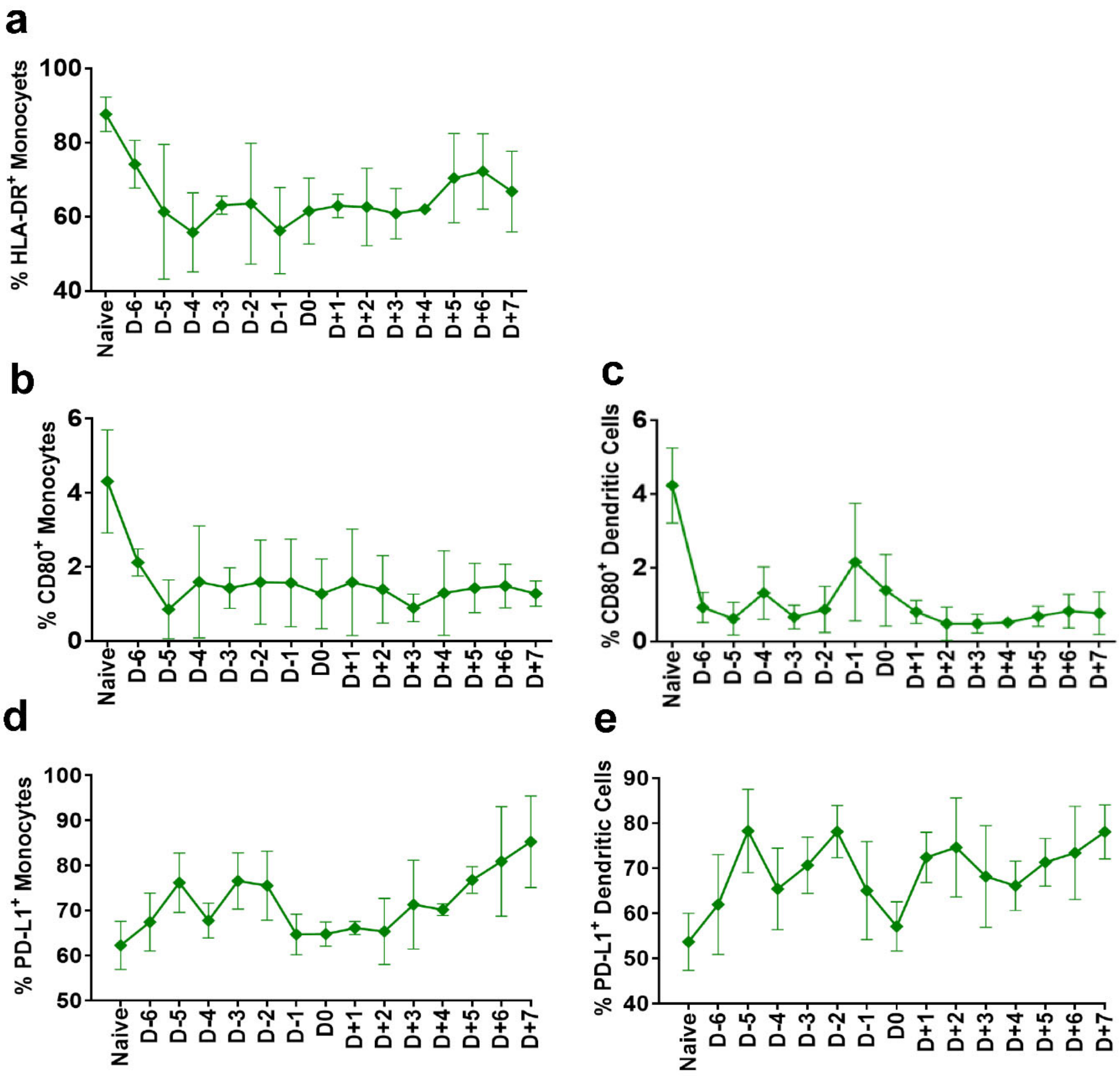

Figure 1. ADL induces tolerogenic APCs. ADL administration in immunosuppressed Cohort A monkeys showed (**a**) substantial reduction in the circulating frequency of HLA-DR$^+$ monocytes: CD14$^+$CD16$^+$. Profound decrease in costimulatory molecules: the percentages of CD80 expressing (**b**) monocytes: CD14$^+$CD16$^+$, (**c**) dendritic cells: CD11c$^+$CD16$^+$ and upregulation of PD-L1 expressing (**d**) monocytes and (**e**) dendritic cells.

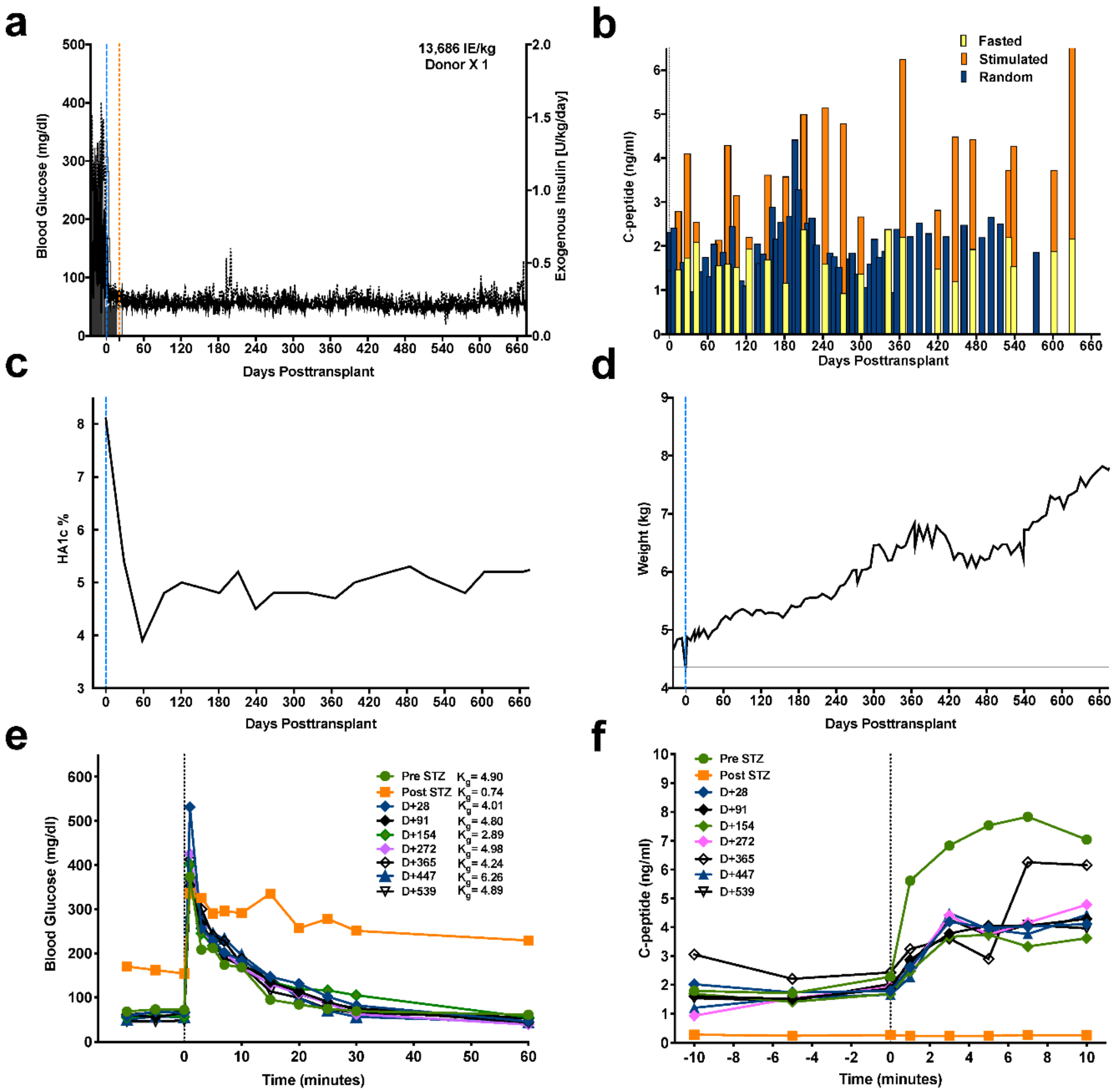

Supplementary Figure 2. Example of long-term (≥22 months) tolerance to islet allograft following peritransplant IV infusions of ADLs under the cover of transient immunosuppression (monkey #15CP1; Cohort C). (**a**) Pre-and postprandial blood glucose (solid and dashed lines, respectively) and daily insulin (bars). (**b**) Positive and stable C- peptide levels (fasted, random, and mixed meal-stimulated) throughout follow-up. (**c**) Restoration of near-normal HbA1c levels throughout follow-up. **(d)** Continued weight gain posttransplant, indicating that posttransplant euglycemia is not due to a malabsorptive state. (**e**) Blood glucose before and after IV infusion of 0.5 g glucose kg^{-1} (IVGTT) and Kg levels before and after diabetes induction and posttransplant. Normal Kg levels posttransplant. (**f**) Acute C-peptide response to IV glucose (0.5 g kg^{-1}).

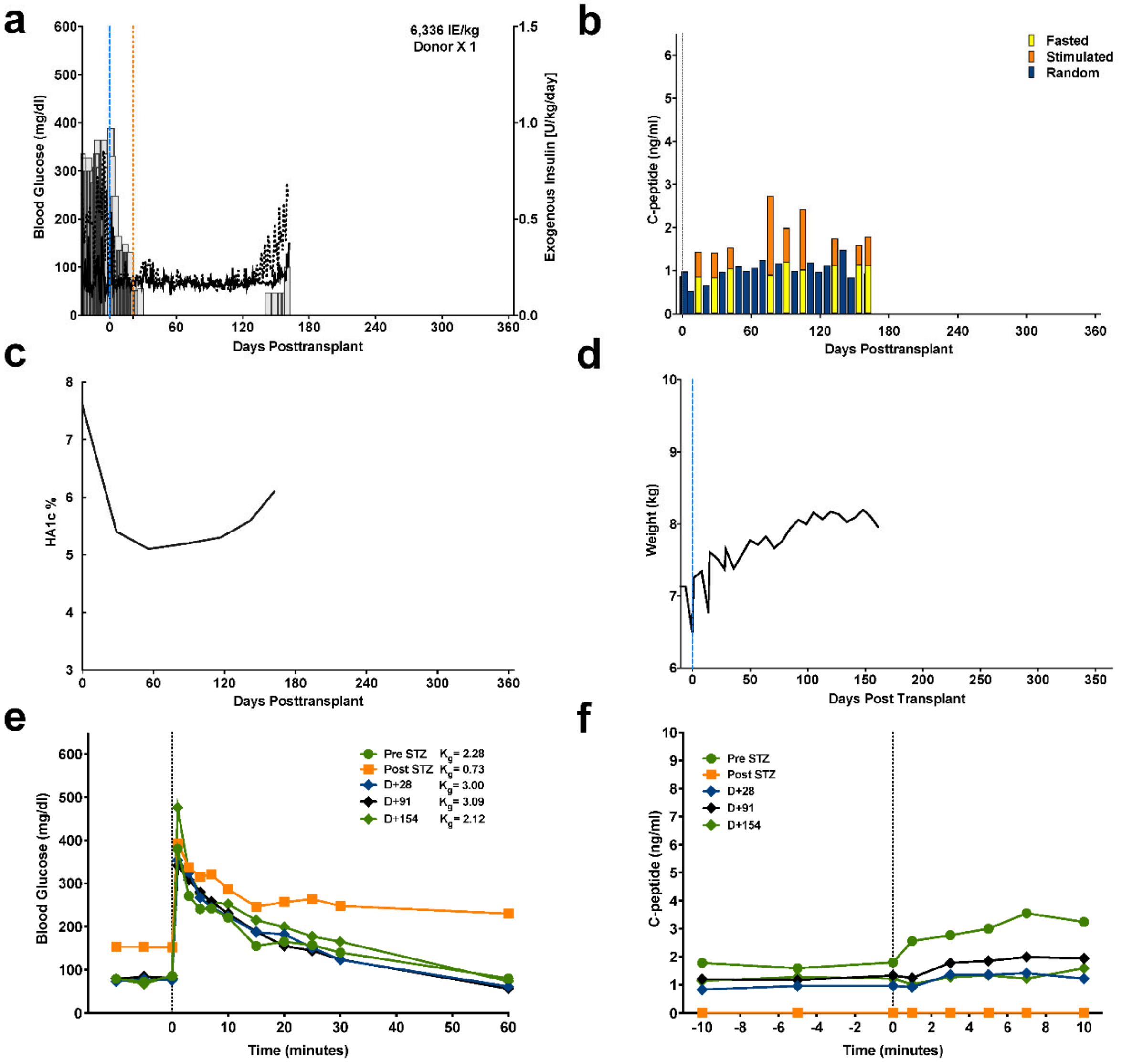

Supplementary Figure 3. Example of islet allograft rejection following transient immunosuppression alone (monkey #15CP3; Cohort B). (**a**) Pre-and postprandial blood glucose levels (solid and dashed lines, respectively) and daily insulin (bars). Postprandial instability was apparent starting day 133 post-transplant with an upward trend, suggestive of allograft loss due to rejection (**b**) C-peptide levels (fasted, random, and mixed meal- stimulated) became positive posttransplant and basal levels remained at approximately 1 ng mL^{-1} through day 161 posttransplant. (**c**) Restoration of near-normal HbA1c levels, increasing around day 140 and trending upward. **(d)** Continued weight gain posttransplant, indicating that posttransplant euglycemia is not due to a malabsorptive state. (**e**) Blood glucose levels before and after IV infusion of 0.5 g glucose kg^{-1} (IVGTT) and Kg levels before and after diabetes induction and post-transplant. Normal Kg levels posttransplant. (**f**) Acute C- peptide response to IV glucose (0.5 g kg^{-1})

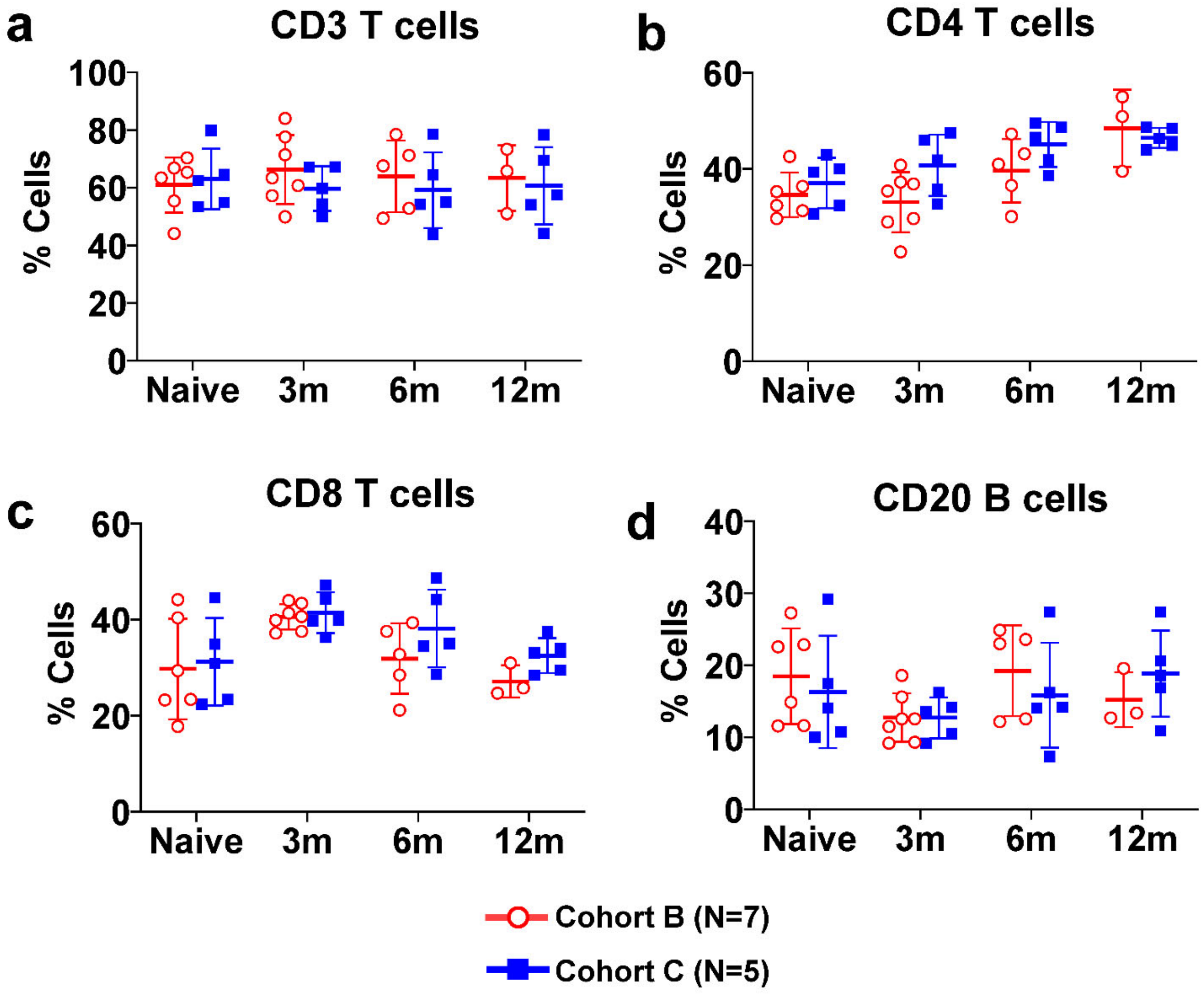

Supplementary Figure 4. Lack of impact of ADL infusions on relative numbers of circulating T and B cells. (**a-d**) Scatter graphs show that relative numbers of various T and B cells remained stable and normalized in the peripheral blood measured longitudinally before and at 3, 6, and 12 months posttransplant in recipients given transient immunosuppression without ADL (Cohort B; n=7; red) and with ADL infusions (Cohort C; n=5; blue). (**a**) Relative numbers of circulating $CD3^+$ T cells. (**b**) Relative numbers of circulating $CD4^+$ T cells. (**c**) Relative numbers of circulating $CD8^+$ T cells. (**d**) Relative numbers of circulating $CD20^+$ B cells.

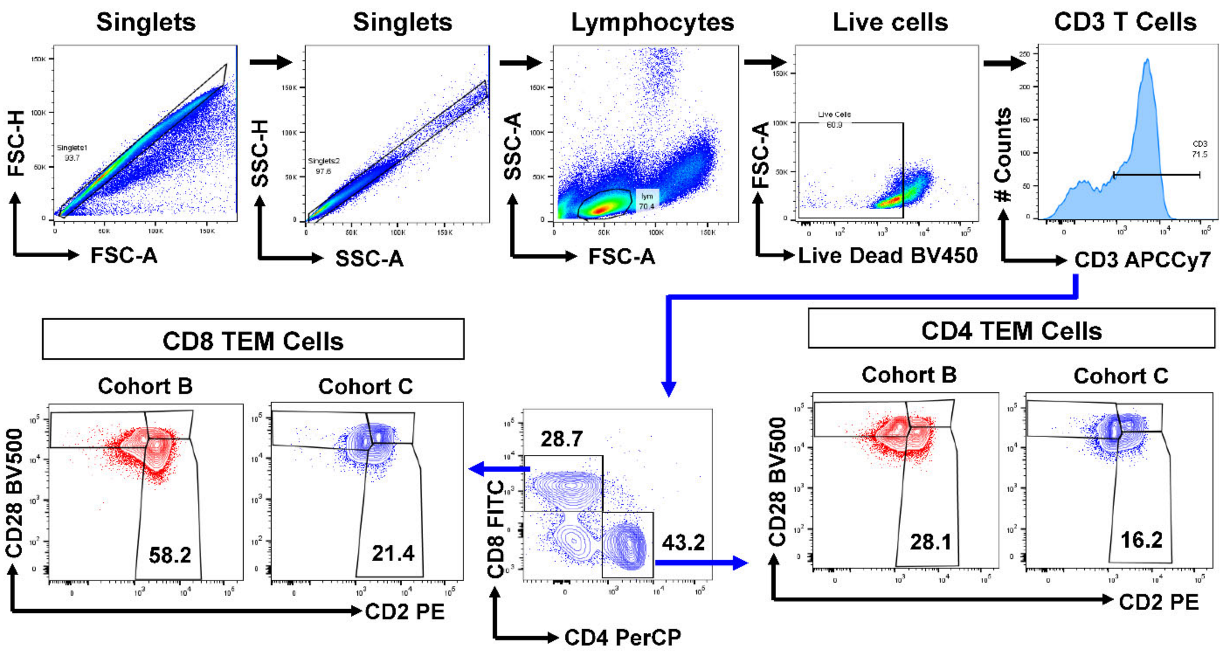

Supplementary Figure 5. Gating strategy for identification of T effector memory (TEM) cell subsets based on CD2 and CD28 expression. Singlets were gated first to eliminate doublets, dead cells were excluded and finally gating was performed on $CD3^{+}CD4^{+}$ and $CD3^{+}CD8^{+}$ to enumerate TEM cells. $CD8^{+}$ (left) and $CD4^{+}$ (right) T cells were further gated based on CD2 and CD28 expression and divided into 3 subsets: $CD2loCD28^{+}$, $CD2hiCD28^{+}$, and $CD2^{hi}CD28^{-}$. Among these, $CD2^{hi}CD28^{-}$ are identified as $CD4^{+}$ and $CD8^{+}$ TEM cells. Representative results from Cohort B (red) and Cohort C (blue) monkeys are shown.

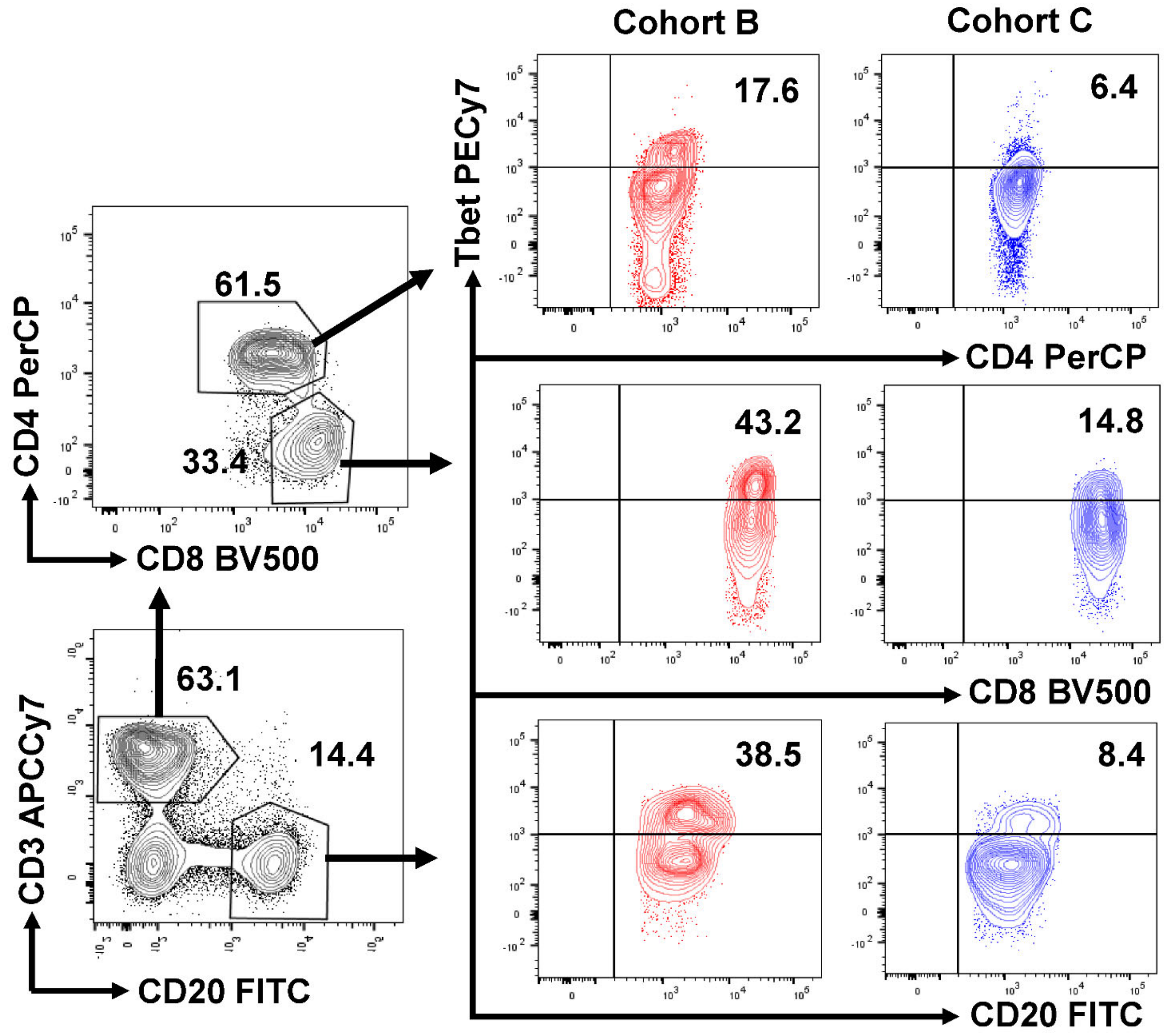

Supplementary Figure 6. Gating strategy for detection of intracellular Tbet expression in T and B cells. Singlets were gated first to eliminate doublets, dead cells were excluded as represented in Supplementary figure 5 and finally gating was performed on $CD3^{+}CD4^{+}$, $CD3^{+}CD8^{+}$ and $CD3^{-}CD20^{+}$ to examine Tbet expression in $CD4^{+}$ T cells (upper row), $CD8^{+}$ T cells (middle row), and $CD20^{+}$ B cells (bottom row), respectively. Representative FACS profiles from Cohort B (red) and Cohort C (blue) monkeys are shown for each subset

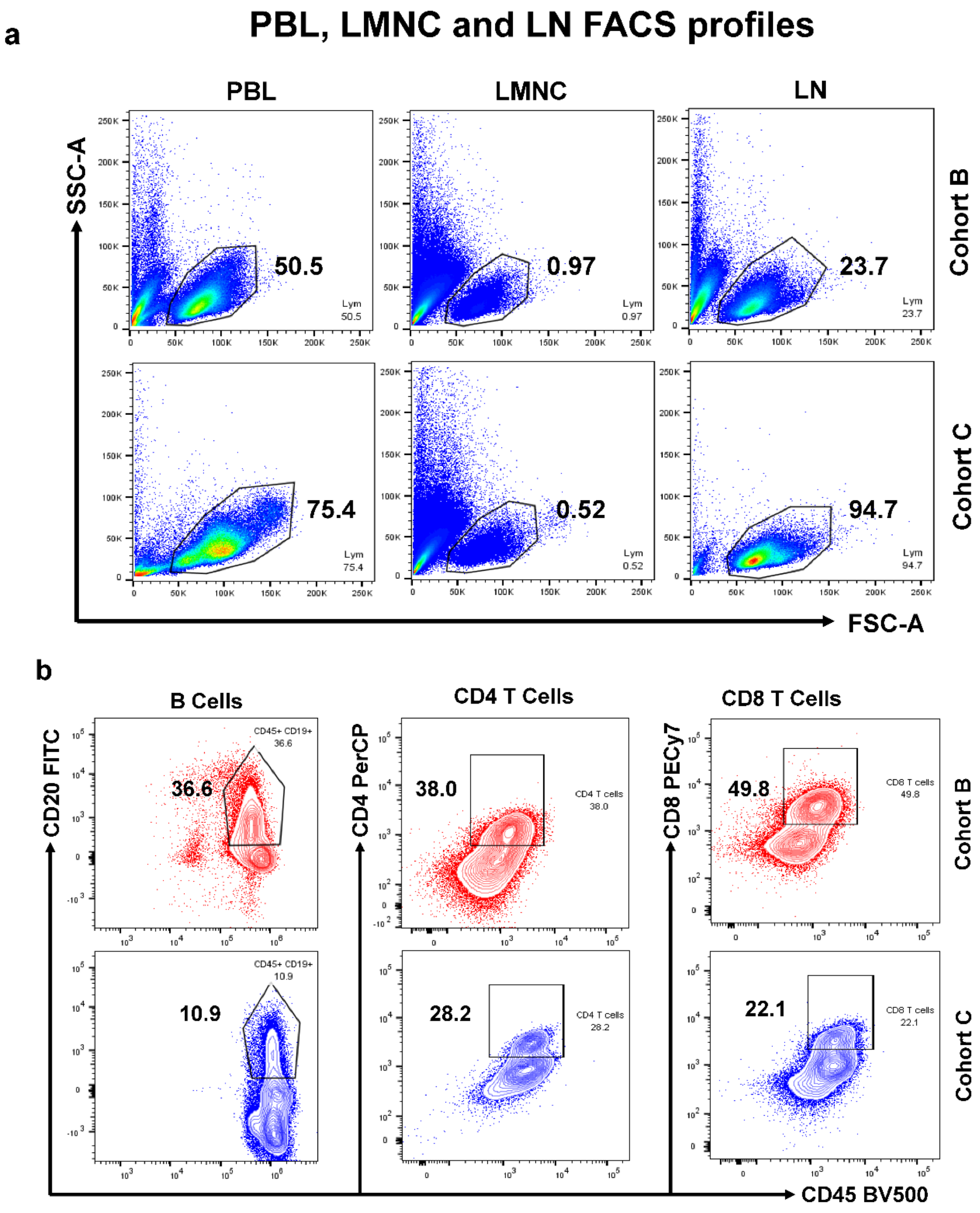

Supplementary Figure 7. Comparative representation of FACS profiles of T and B cells subsets in PBLs, LMNCs, and LNs. (a) Presenting FACS profile on forward and side scatters for cell preparation obtained from PBLs, LMNCs, and LNs both in Cohort B vs Cohort C monkeys. (**b**) FACS plots show how CD20^{+} B cells as well as CD4^{+} and CD8^{+} T cells were gated on CD45^{+} lymphocytes both in Cohort B (red) and Cohort C (blue) monkeys.

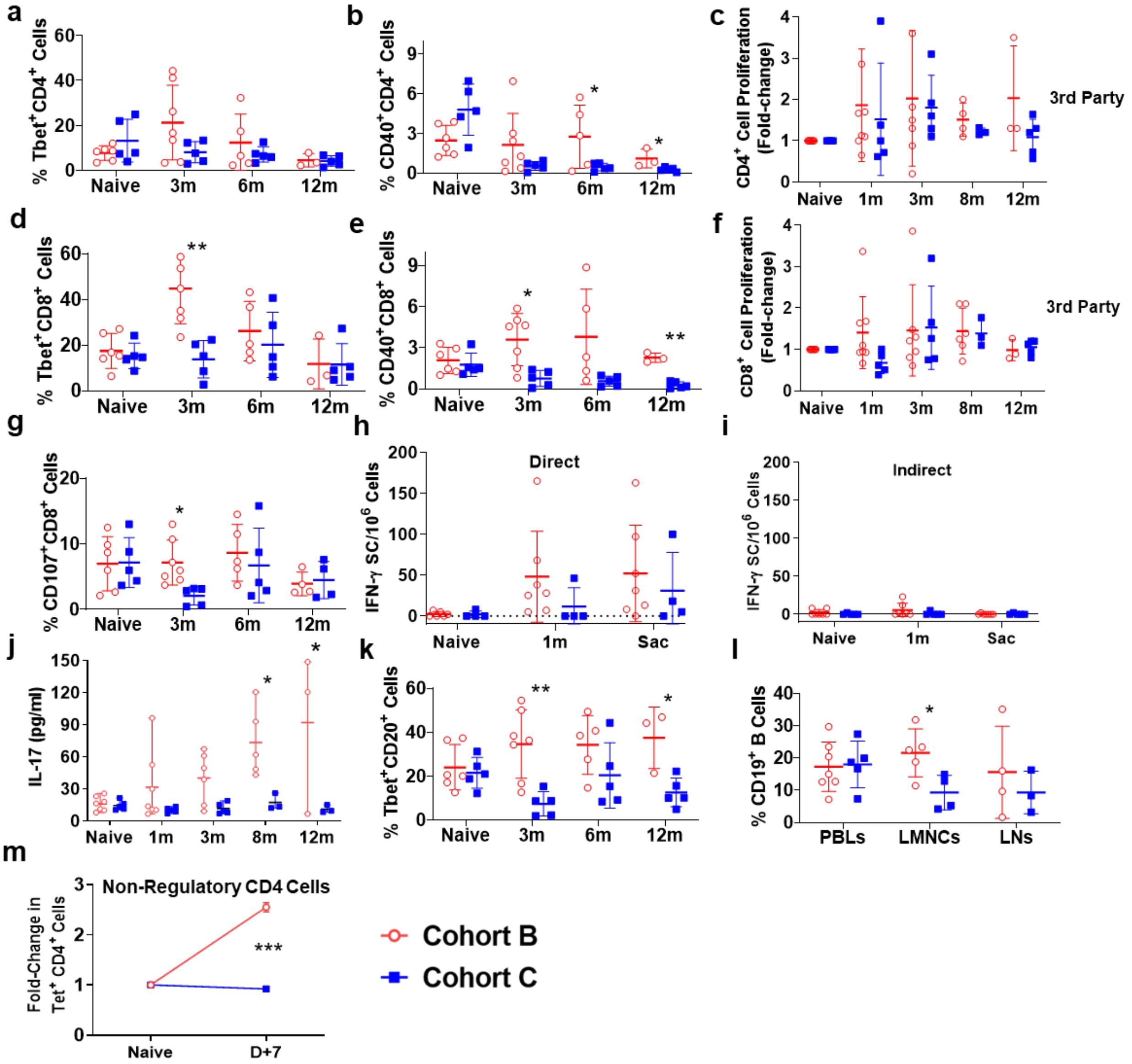

Supplementary Figure 8. Effector T and B cell subsets. T and B immune subsets in the peripheral blood measured before and at 3, 6, and 12 months posttransplant and at time of termination in recipients given transient immunosuppression without ADL (Cohort B; n=7; red) and with ADL infusions (Cohort C; n=5; blue). (**a**) Relative numbers of circulating CD4$^+$ Tbet$^+$ (% Tbet$^+$ CD4$^+$ Cells), (b) Th40 cells (% CD40$^+$ CD4$^+$ Cells) and (**c**) Fold-Change in proliferation of CFSE-labeled CD4$^+$ T in response to irradiated third-party PBLs before and at the indicated intervals posttransplant in a 6-day MLR. (**d**) Relative numbers of circulating CD8$^+$ Tbet$^+$ (% Tbet$^+$ CD8$^+$ Cells), (e) Th40 cells (% CD40$^+$ CD8$^+$ Cells) and (**f**) Fold-Change in proliferation of CFSE-labeled CD8$^+$ T cells in response to irradiated third-party PBLs before and at the indicated intervals posttransplant in a 6-day MLR. (**g**) Circulatory frequency of CD107a$^+$CD8$^+$ T cells. (**h-i**) Frequency of circulating IFN-γ secreting T cells (SC) – with direct donor specificity (Direct; **h**) and indirect donor specificity (Indirect; **i**) in one-way ELISPOT–the frequency of circulating IFN-γ

secreting T cells is presented as scatter plots. * $P < 0.05$; one-way Student's t test. (**j**) ADL infusions significantly suppressed IL-17 protein levels in supernatants of donor-stimulated PBLs collected at intervals posttransplant from Cohort C recipients when compared with IL-17 levels in posttransplant Cohort B MLRs. (**k-l**) Relative numbers of circulating Tbet$^+$ B cells before and at 3, 6, and 12 months posttransplant (**k**) in PBLs and CD20$^+$ B cells (**l**) in PBLs, LMNCs, and LNs cells at time of termination in islet allograft recipients from Cohort B (n=4 to 7, red) and Cohort C (n= 3 to 5, blue) (**l**) Fold-Change in percentage of circulating non-regulatory CD4$^+$ T cells with indirect donor MHC class I specificity among Cohort B (n=3) and C (n=2) monkeys. *$P < 0.05$ and **$P <$ 0.001, ***$P < 0.001$, Student's t test (panels l), non-parametric Mann-Whitney U test followed by post-hoc analysis with the Holm-Sidak method (panel m) and non-parametric Wilcoxon Rank Sum test (all other panels).

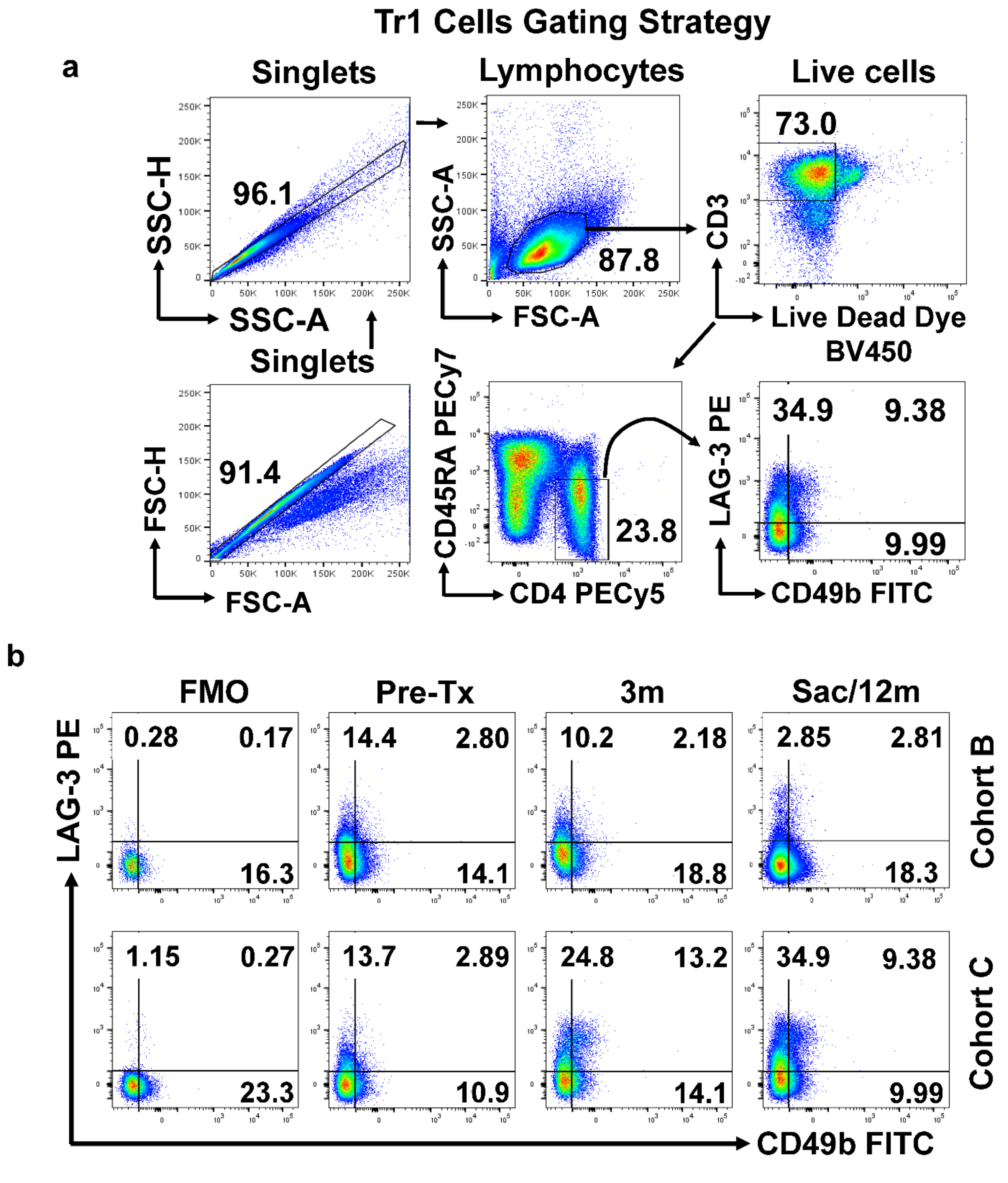

Supplementary Figure 9. Flow gating strategy for Tr1 cells. (**a**) Tr1 cells ($CD49b^{+}LAG\text{-}3^{+}$ gated on $CD4^{+}CD45RA^{-}$ of $CD3^{+}$ T cells) excluding doublets and dead cells. (**b**) Representation of Tr1 cells at pretransplant, 3 months posttransplant, and either 12 months posttransplant or time of termination from Cohort B (top row) and Cohort C (bottom row) recipient monkeys, shown with fluorescence minus one (FMO) control for LAG-3 staining.

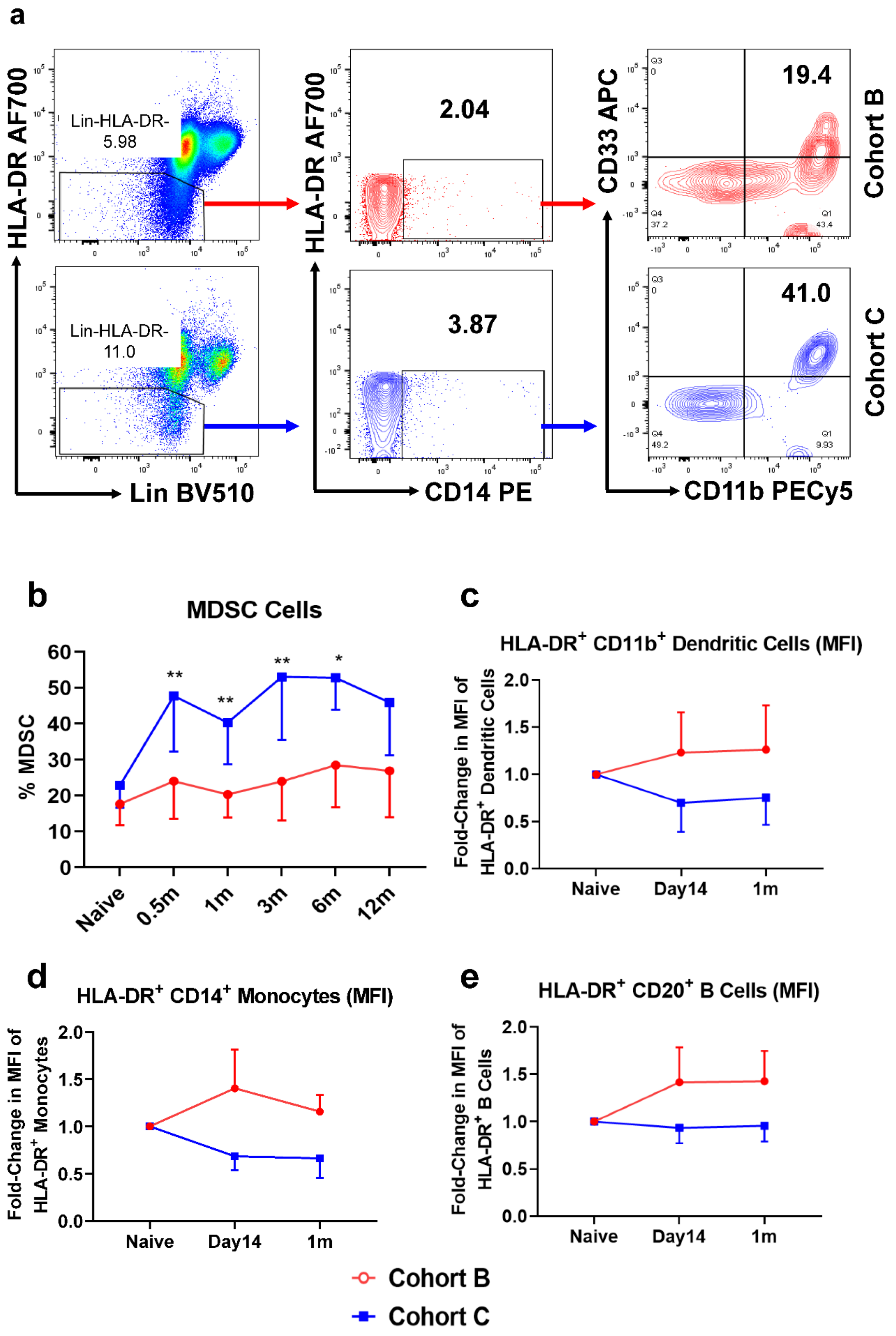
a
Lin-HLA-DR-
5.98
Lin-HLA-DR-
11.0
HLA-DR AF700
Lin BV510
2.04
3.87
HLA-DR AF700
CD14 PE
19.4
41.0
Q3
0
Q4
37.2
Q1
43.4
Q4
49.2
Q1
9.93
CD33 APC
CD11b PECy5
Cohort B
Cohort C
b
MDSC Cells
% MDSC
Naive
0.5m
1m
3m
6m
12m
c
HLA-DR+ CD11b+ Dendritic Cells (MFI)
Fold-Change in MFI of HLA-DR+ Dendritic Cells
Naive
Day14
1m
d
HLA-DR+ CD14+ Monocytes (MFI)
Fold-Change in MFI of HLA-DR+ Monocytes
e
HLA-DR+ CD20+ B Cells (MFI)
Fold-Change in MFI of HLA-DR+ B Cells
Cohort B
Cohort C

Supplementary Figure 10. ADL induces expansion of MDSC and tolerogenic APCs. (**a**) Gating strategy for identification of MDSC. Singlets were gated first to eliminate doublets and dead cells were excluded. Based on $CD33^+$ and $CD11b^+$ coexpression, MDSCs were identified in gated $CD14^+$ cells within the $Lin^-HLA\text{-}DR^-$ population and with Lin^- depicting $CD3^-CD20^-$ cells. Representative FACS profiles from one Cohort B (Red) and one Cohort C (Blue) monkeys are shown. (**b**) Significant increase in percentage of circulating MDSC among Cohort C monkey compared to Cohort B monkey. Fold-change in MFI of circulating (**c**) $HLA\text{-}DR^+$ $CD11b^+$ dendritic cells, (**d**) $HLA\text{-}DR^+$ $CD14^+$ monocytes and (**e**) $HLA\text{-}DR^+$ $CD20^+$ B cells among Cohort B and C monkeys. $^*P < 0.01$, $^{**}P < 0.001$, non-parametric Mann-Whitney U test followed by post-hoc analysis with the Holm-Sidak method.

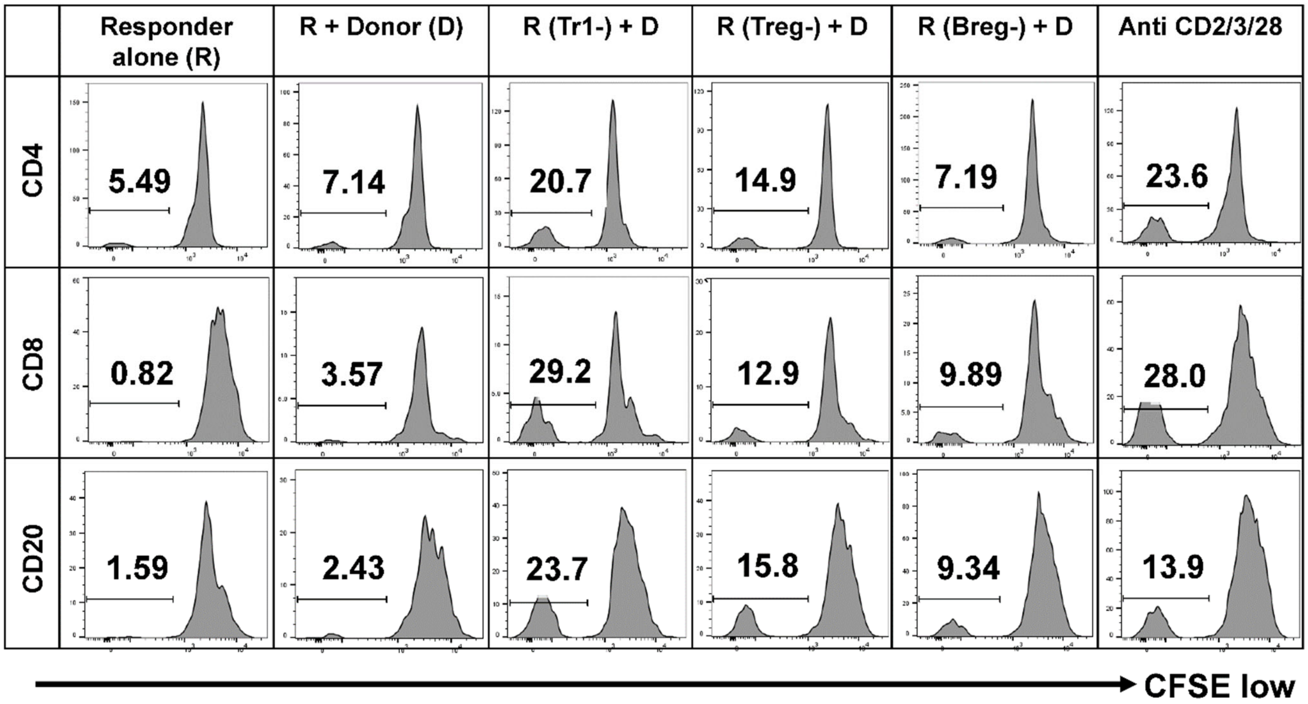

Supplementary Figure 11. Impact of depletion of regulatory cells on donor specific proliferation of T and B cells. Representative FACS plots showing percentages of T and B cells proliferating (CFSE low) under different experimental conditions. Tr1-, Treg-, Breg- depict responder PBLs depleted of Tr1, Treg and Breg cells.

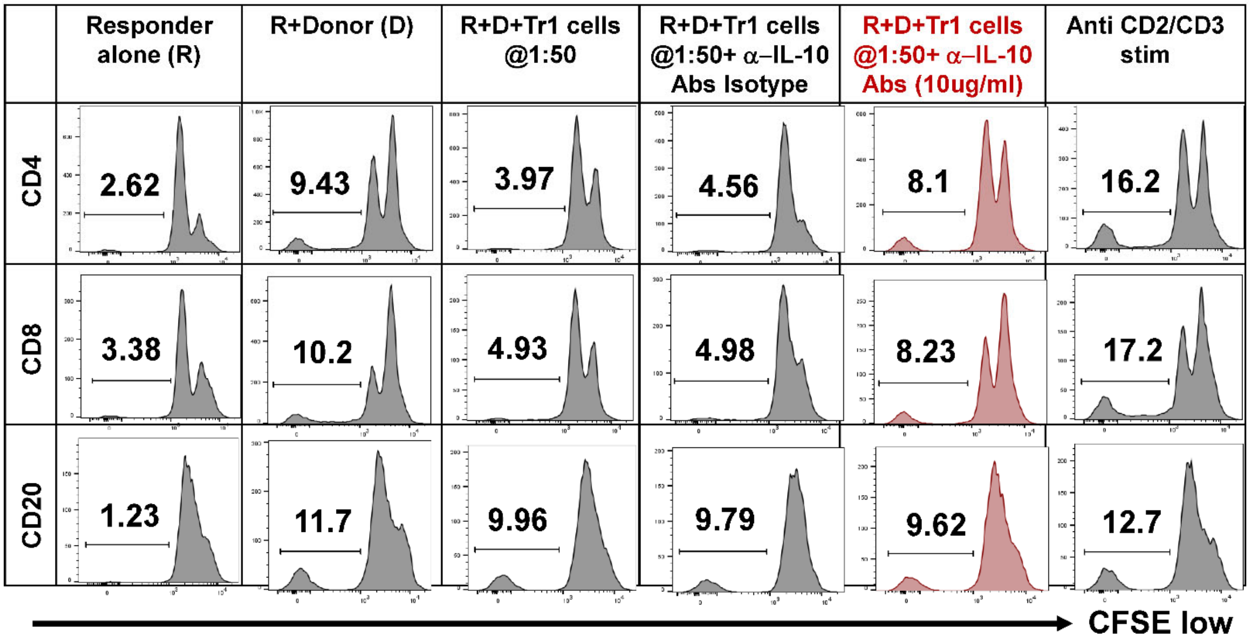

Supplementary Figure 12. IL-10 neutralization substantially abrogates Tr1 cell- mediated suppression of T and B cell proliferation. Representative FACS plots shows percentage of Tr1 cell-depleted responder T and B cells proliferating (CFSE low) under different experimental conditions.

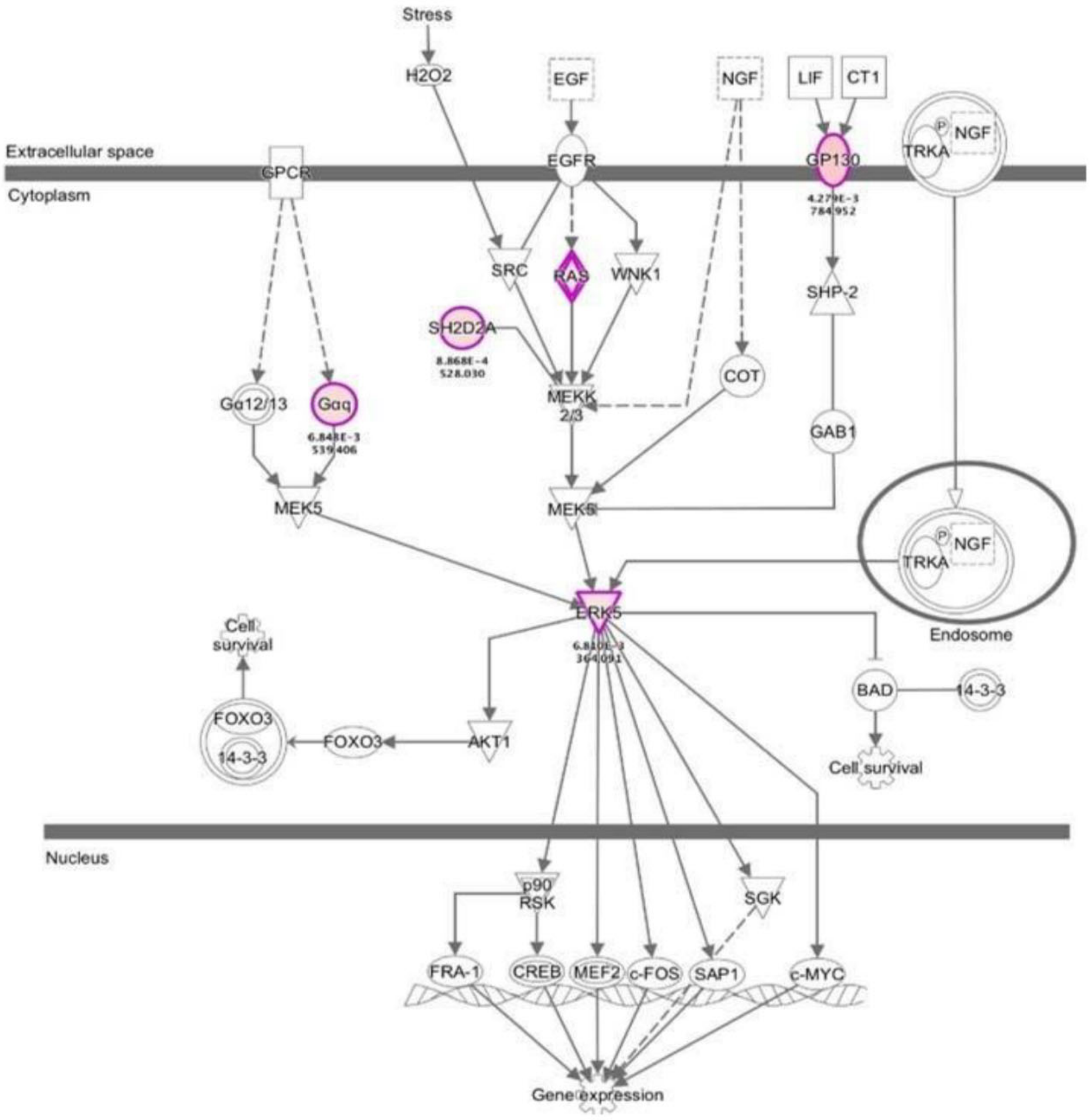

Supplementary Figure 13. ERK5 signaling cascade identified by pathway analysis. Pathway analysis of differentially expressed genes identified ERK5 signaling as one of the major pathways activated in Tr1 cells retrieved from Cohort C monkeys. The differentially expressed genes are colored.

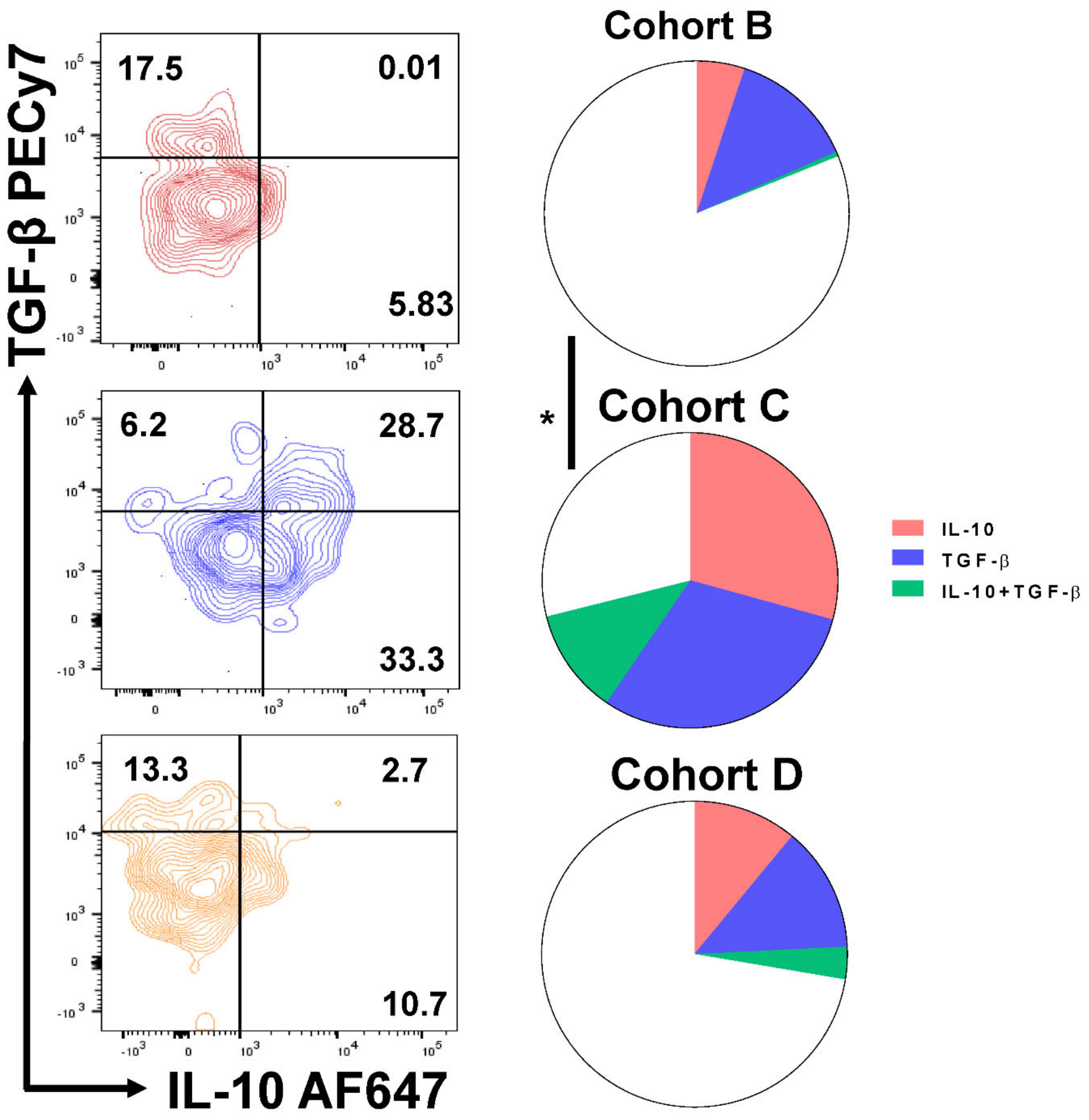

Supplementary Figure 14. Suppressive cytokines IL-10 and TGF-β production by donor stimulated Tr1 cells. Boolean gating of donor-specific Tr1 cells reveals three distinct cytokine-producing populations. Representative FACS plots shows the percentage of Tr1 cells and pie chart shows total proportion of Tr1 cells producing the indicated cytokines among Cohort B (n=3), Cohort C (n=3), and fully MHC mismatched Cohort D (n=2) monkeys. For pie charts, red, blue and green arcs represent total proportion of Tr1 cells producing IL-10, TGF-β, and both IL-10+TGF-β.

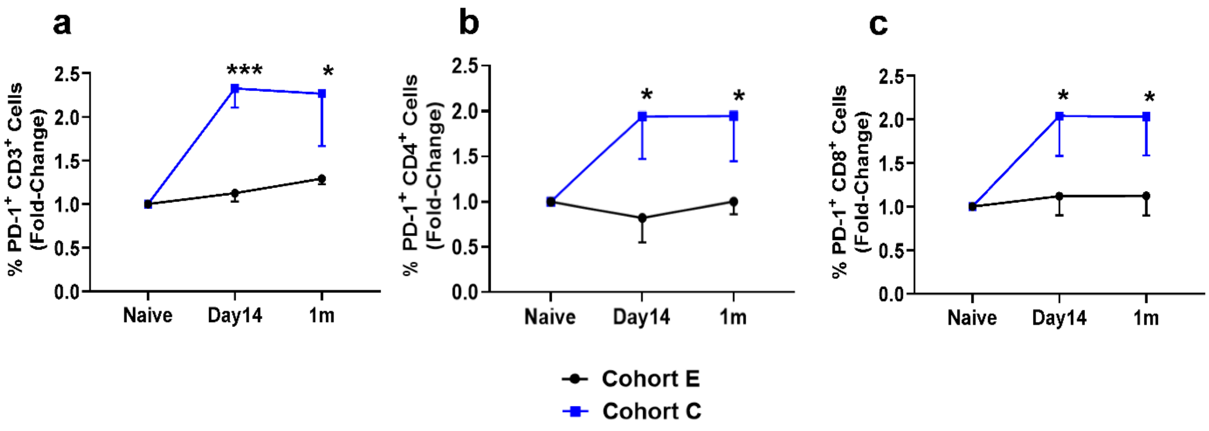

Supplementary Figure 15. ADL administration induces exhausted T cell phenotype only in nonsensitized Cohort C monkeys. Significant fold-change in PD-1 expressing (**a**) $CD3^+$ T cells (**b**) $CD4^+$ T cells and (**c**) $CD8^+$ T cells among Cohort C monkey (nonsensitized, blue) compared to Cohort E monkey (sensitized, black).

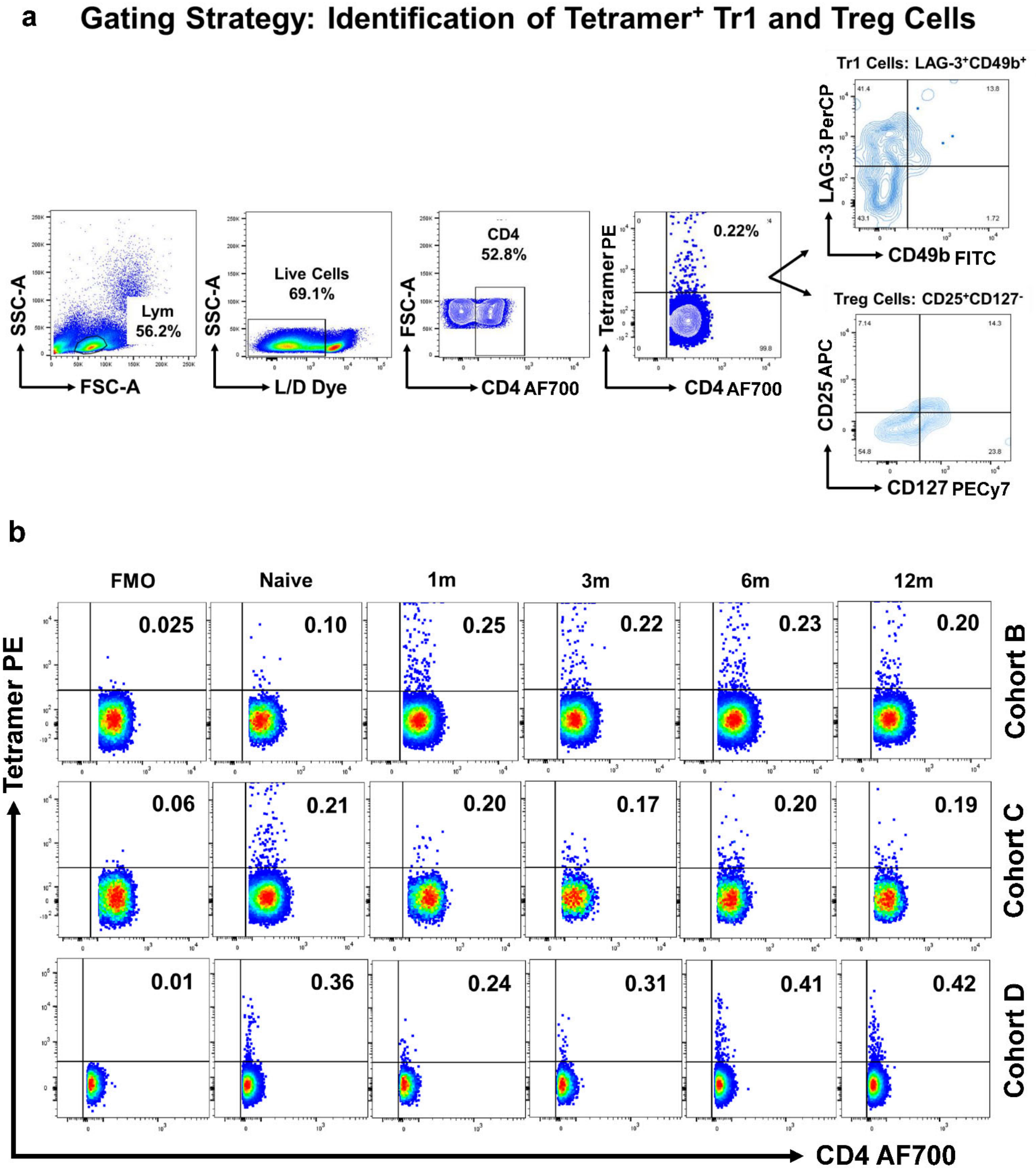

a
Gating Strategy: Identification of Tetramer+ Tr1 and Treg Cells
Lym
56.2%
SSC-A
FSC-A
Live Cells
69.1%
SSC-A
L/D Dye
CD4
52.8%
FSC-A
CD4 AF700
Tetramer PE
0.22%
CD4 AF700
Tr1 Cells: LAG-3+CD49b+
LAG-3 PerCP
CD49b FITC
41.4
13.8
43.1
1.72
Treg Cells: CD25+CD127-
CD25 APC
CD127 PECy7
7.14
14.3
54.8
23.8
b
FMO
Naive
1m
3m
6m
12m
Cohort B
0.025
0.10
0.25
0.22
0.23
0.20
Cohort C
0.06
0.21
0.20
0.17
0.20
0.19
Cohort D
0.01
0.36
0.24
0.31
0.41
0.42
Tetramer PE
CD4 AF700

c

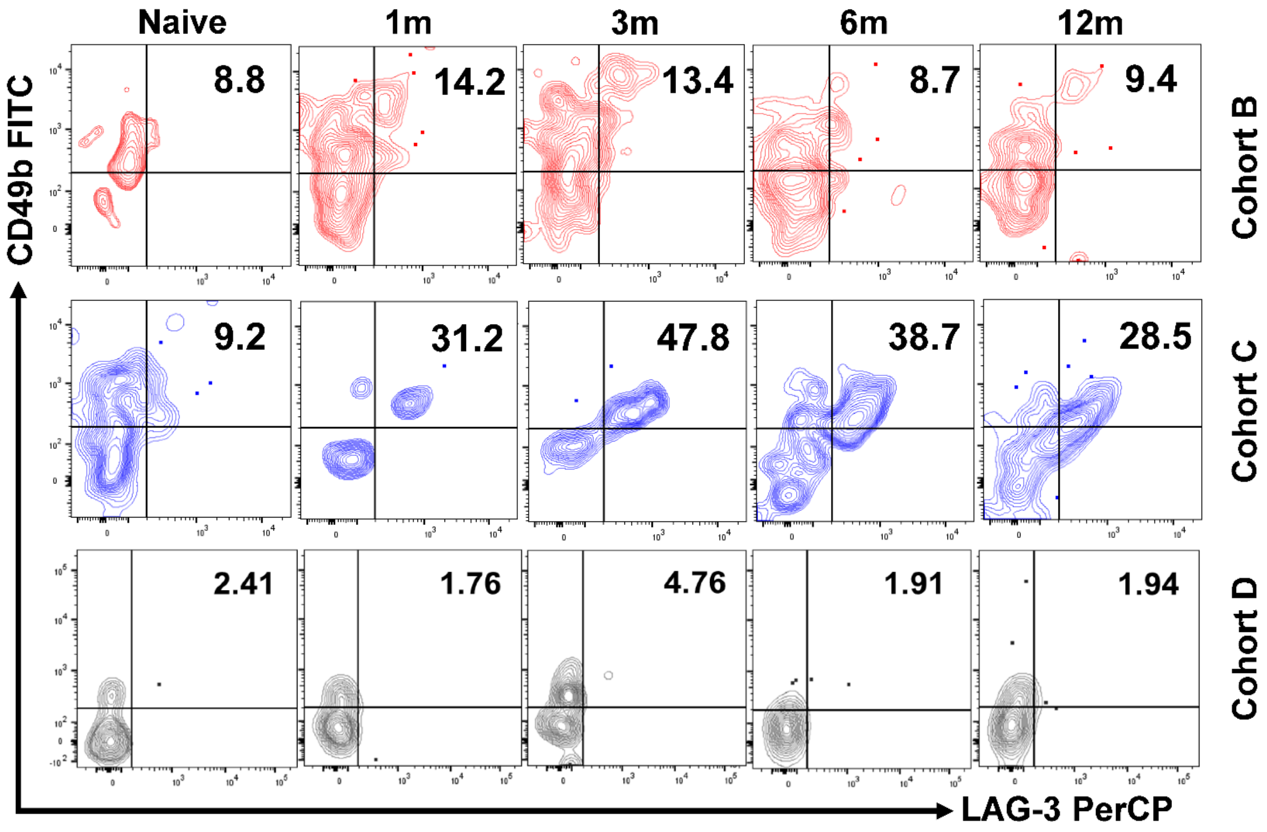
Naive
1m
3m
6m
12m
8.8
14.2
13.4
8.7
9.4
Cohort B
9.2
31.2
47.8
38.7
28.5
Cohort C
2.41
1.76
4.76
1.91
1.94
Cohort D
CD49b FITC
LAG-3 PerCP

d

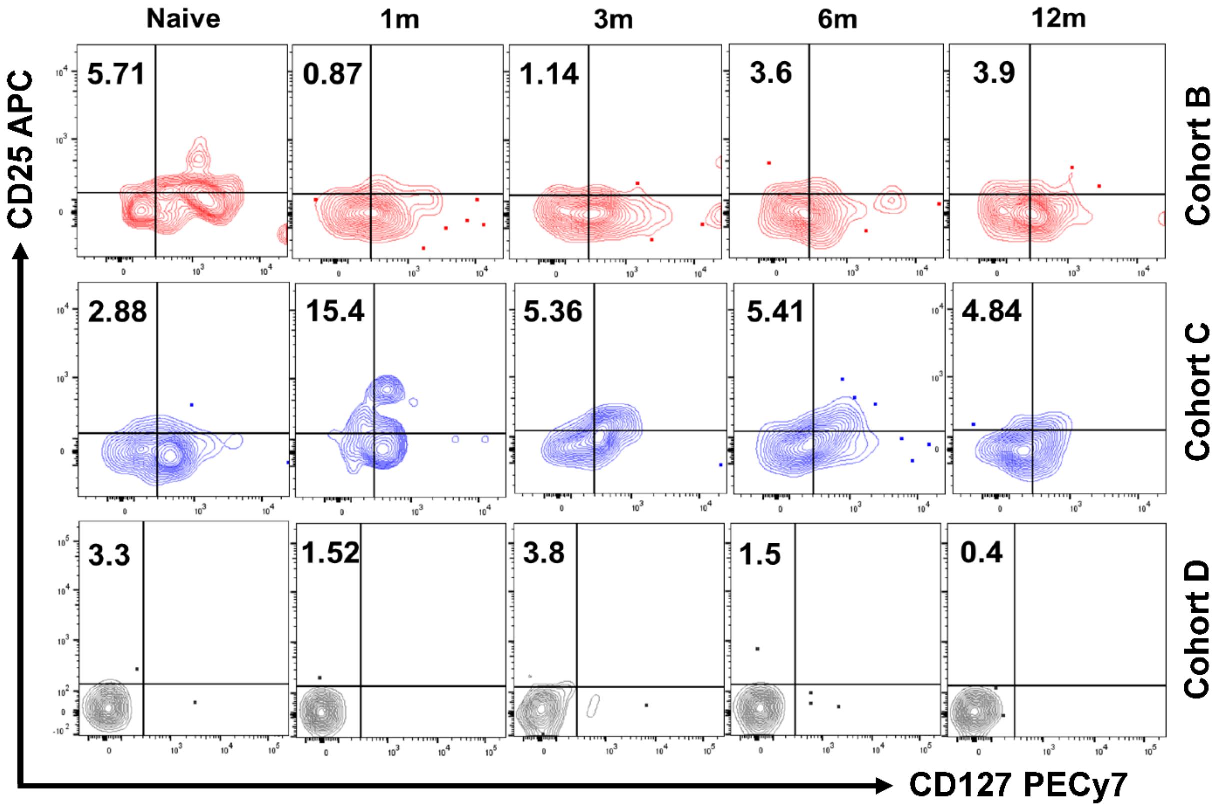
Naive
1m
3m
6m
12m
5.71
0.87
1.14
3.6
3.9
Cohort B
2.88
15.4
5.36
5.41
4.84
Cohort C
3.3
1.52
3.8
1.5
0.4
Cohort D
CD25 APC
CD127 PECy7

Supplementary Figure 16. Flow gating strategies for tetramer staining. (**a**) Flow gating strategy showing enumeration of tetramers^{+} total CD4 T cells, Tr1 and Treg cells. (**b**) The percentages of tetramer^{+} CD4^{+} T cells are shown in each plot at indicated time points in the circulation from each cohort. FMO (Fluorescence Minus One) was used to set up the threshold gate. Similarly, non-regulatory tetramer^{+}CD4^{+} T cells were identify after excluding CD25^{+},CD49b^{+} and LAG-3^{+} cells within gated CD4 lym. (**c**) Percentage of Tr1 cells (CD49b^{+}LAG-3^{+}) and (**d**) Percentage of Treg cells (CD25^{+}CD127^{-}) within gated tetramer^{+} CD4^{+} lym among Cohort B, C, and D monkeys.

Supplementary Table 1. Cohorts of recipient monkeys studied. The table indicates the number of animals studied in each cohort, their ID number, diabetes status, their treatment with islet allografts, their treatment with ADL infusions, their DRB matching status, their treatment with immunosuppression, their baseline Class I and II DSA status, and their baseline anti-donor IFN-γ ELISPOT. Non-sensitized: <2500 mfi in flow cross-match and <44 IFN-γ secreting cells in direct ELISPOT. Sensitized: >2500 mfi in flow cross-match) and >44 IFN-γ secreting cells in direct ELISPOT.

Cohorts	n	ID	STZ-Induced Diabetes	Islet Transplant	ADL Infusions	One DRB-Matched D/R Pairs	Immuno-Suppr.	Base line DSA (MFI)		Base line ELISPOT (IFN-γ SC/10^6)
								Class I	Class II	
A Exploratory	3	17EP18	No	No	Yes	Yes	Yes	391	376	2
		17EP6	No	No	Yes	Yes	Yes	232	66	0
		17EP3	No	No	Yes	Yes	Yes	177	256	0
B Control (w/o ADL)	7	13EP12	Yes	Yes	No	Yes	Yes	225	415	1
		13EP8	Yes	Yes	No	Yes	Yes	248	1358	5
		14HP34	Yes	Yes	No	Yes	Yes	187	414	15
		14HP26	Yes	Yes	No	Yes	Yes	281	1929	2
		15CP3	Yes	Yes	No	Yes	Yes	222	247	0
		14HP31	Yes	Yes	No	Yes	Yes	173	321	2
		15CP6	Yes	Yes	No	Yes	Yes	223	468	0
C Experimental (with ADL)	5	13EP5	Yes	Yes	Yes	Yes	Yes	188	244	0
		14HP33	Yes	Yes	Yes	Yes	Yes	282	1071	0
		14HP24	Yes	Yes	Yes	Yes	Yes	201	397	3
		15CP4	Yes	Yes	Yes	Yes	Yes	238	225	0
		15CP1	Yes	Yes	Yes	Yes	Yes	232	732	0
D Experimental Mismatched	3	15FP01	Yes	Yes	Yes	No	Yes	384	396	18
		15FP02	Yes	Yes	Yes	No	Yes	495	782	16
		15FP03	Yes	Yes	Yes	No	Yes	450	509	10
E Sensitized	4	15FP13	Yes	Yes	No	Yes	Yes	284	5402	8
		14HP21	Yes	Yes	Yes	Yes	Yes	525	2675	15
		14HP29	Yes	Yes	Yes	Yes	Yes	607	2744	0
		13EP03	Yes	Yes	Yes	Yes	Yes	645	1676	73

Supplementary Table 2. Mapped TCRβ clonotypes in the Cohort A.

V	D	J
V11-1*01	D1*01	J2-7*01
V11-2*01	D1*01	J2-3*01
V11-3*01	D2*01	J2-7*01
V12-2*01	D1*01	J2-4*01
V12-2*01	D1*01	J1-5*01
V12-2*01,V12-3*01		J2-1*02,J2-1*01
V12-2*01,V12-3*01	D2*01,D1*01	J2-3*01
V12-2*01,V12-3*01	D2*01,D1*01	J2-3*01
V13*01	D1*01	J2-2*01
V14*01	D2*01,D1*01	J2-2*01
V19*01		J2-6*01
V20-1*01	D1*01	J2-1*02,J2-1*01
V2-1*01	D1*01	J2-7*01
V21-1*01	D2*01	J1-4*01
V2-2*01,V2-3*01		J2-5*01
V2-3*01	D2*01	J2-3*01
V23-1*01	D2*01	J2-7*01
V24-1*01	D1*01,D2*01	J2-1*02,J2-1*01
V27*01	D1*01	J2-3*01
V28*01	D1*01	J1-5*01
V28*01	D2*01	J2-5*01
V28*01		J2-5*01
V29-1*01	D2*01	J2-1*02,J2-1*01
V3-2*01,V3-1*01	D1*01	J2-1*02,J2-1*01
V4-2*01	D1*01	J2-2*01
V4-2*01	D2*01	J2-2*01
V4-2*01		J1-4*01
V4-2*01		J1-6*02,J1-6*01
V5-1*01	D1*01,D2*01	J1-4*01
V5-1*01	D1*01	J1-2*01
V5-6*01	D1*01	J2-3*01

Supplementary Table 3. Weight of the recipient monkeys.

Group	Animal ID	Baseline Weight	Endpoint Weight	Endpoint Weight (PM)
A	Nebula 17EP6	13.64	13.02	13.44
A	Amazo 17EP18	10.81	10.55	10.75
A	Rocket 17EP3	13.05	12.60	13.28
B	Tiki 13EP12	6.53	7.92	8.44
B	Saga 13EP8	5.15	7.18	7.40
B	Renly 14HP31	5.42	6.40	7.49
B	Frey 14HP26	5.40	6.34	7.35
B	Grivas 14HP34	3.60	3.36	3.77
B	Dalby 15CP3	6.50	7.31	7.96
B	Tully 15CP6	8.95	12.20	13.09
C	Fitch 13EP5	6.67	8.88	9.29
C	Sansa 14HP33	5.24	5.25	6.12
C	Tambo 15CP1	4.36	8.09	8.82
C	Coen 15CP4	6.60	8.87	9.93
C	Borcus 14HP24	3.49	4.61	5.49
D	Atik 15FP1	4.57	6.21	7.12
D	Castor 15FP2	5.29	5.58	6.92
D	Nash 15FP3	5.70	7.30	7.69
E	Baldur 13EP3	6.15	7.31	7.69
E	Stark 14HP21	5.71	5.92	6.28
E	Tyrion 14HP29	3.73	3.87	4.35
E	Bowen 15FP13	8.28	8.40	9.23

Supplementary Table 4. Graft histopathology of Cohort C monkey #13EP5.

Slide	*A*	*B*	*C*	*D*	*E*	*F1*	*F2*
#1	6	0	0	0	0	0	0
#2	3	2	0	0	0	0	0
#3	1	3	0	0	0	0	0
#4	7	10	1	0	0	0	0
#5	6	3	0	0	0	0	0
#6	10	2	0	0	0	0	0
#7	4	2	0	0	0	0	0
#8	6	2	0	0	0	0	0
#9	6	4	0	0	0	0	0
#10	3	3	0	0	0	0	0
**Totals**	**52**	**31**	**1**	**0**	**0**	**0**	**0**

Key to Table

A = Normal islet
B = Mild lymphocytic infiltrate surrounding islet
C = Moderate lymphocytic infiltrates with infiltration
D = Marked infiltrates with extensive loss of islet cells
E = Obliteration of islet with lymphocytic ± other inflammatory cell infiltrates as noted
F1 = Partial islet cell loss with fibrosis; no/minimal inflammation
F2 = Complete islet cell loss with fibrosis; no/minimal inflammation

Supplementary Table 5. Graft histopathology of Cohort B monkey # 15CP1.

Slide	*A*	*B*	*C*	*D*	*E*	*F1*	*F2*
#1	4	0	0	0	0	0	0
#2	1	0	0	0	0	0	0
#3	8	0	0	0	0	0	0
#4	7	0	0	0	0	0	0
#5	3	0	0	0	0	0	0
#6	7	0	0	0	0	0	0
#7	9	1	0	0	0	0	0
#8	3	1	0	0	0	0	0
#9	4	0	0	0	0	0	0
#10	0	0	0	0	0	0	0
**Totals**	**46**	**2**	**0**	**0**	**0**	**0**	**0**

Key to Table
A = Normal islet
B = Mild lymphocytic infiltrate surrounding islet
C = Moderate lymphocytic infiltrates with infiltration
D = Marked infiltrates with extensive loss of islet cells
E = Obliteration of islet with lymphocytic ± other inflammatory cell infiltrates as noted
F1 = Partial islet cell loss with fibrosis; no/minimal inflammation
F2 = Complete islet cell loss with fibrosis; no/minimal inflammation

Supplementary Table 6. Histopathology of the native pancreas at necropsy.

Animal ID	Cohort	Native pancreas Histology
13EP12	B	Two sections of pancreas were examined. Islets appeared normal in size to slightly smaller than normal and many islet cells were smaller than normal. A few scattered insulin positive cells were present in the islets and in the exocrine pancreas.
13EP8	B	One section of pancreas was examined. Islets appeared normal in size to slightly smaller than normal. Many islet cells were smaller than normal with reduced and minimally vacuolated cytoplasm. A few scattered insulin positive cells were present in the islets and in the exocrine pancreas.
14HP26	B	One section of pancreas was examined. Islets appeared normal in size to slightly smaller than normal and many islet cells were smaller than normal. Rare scattered insulin positive cells were present in the islets and in the exocrine pancreas.
14HP31	B	Three sections of pancreas were examined. Islets appeared normal in size to slightly smaller than normal. Many islet cells were smaller than normal with reduced and minimally vacuolated cytoplasm. A few insulin positive cells were present in the islets and in the exocrine pancreas.
14HP34	B	One section of pancreas was examined. Islets appeared smaller than normal and many islet cells were smaller than normal. Rare scattered insulin positive cells were present in the islets and in the exocrine pancreas.
15CP3	B	Three sections of pancreas were examined. Islets appeared normal in size to slightly smaller than normal. Most islet cells were smaller than normal with reduced cytoplasm. No insulin positive cells were present.
15CP6	B	One section of pancreas was examined. Islets appeared normal in size to moderately smaller than normal. Many islet cells were smaller than normal with reduced and minimally vacuolated cytoplasm. Rare insulin positive cells were present in the islets and in the exocrine pancreas.
13EP5	C	One section of pancreas was examined. Islets appeared normal in size to slightly smaller than normal and many islet cells were smaller than normal. A few scattered insulin positive cells were present in the islets and in the exocrine pancreas.
14HP24	C	One section of pancreas was examined. Islets appeared slightly to moderately smaller than normal. Many islet cells were smaller than normal with reduced and minimally vacuolated cytoplasm. Rare insulin positive cells were present in the islets or exocrine pancreas.
14HP33	C	Two sections of pancreas were examined. Islets appeared normal in size to slightly smaller than normal. Many islet cells were smaller than normal with reduced and minimally vacuolated cytoplasm. A few insulin positive cells were present in the islets and in the exocrine pancreas.
15CP1	C	One section of pancreas was examined. Islets appeared slightly to moderately smaller than normal. Many islet cells were smaller than normal with reduced and minimally vacuolated cytoplasm. Rare insulin positive cells were present in the islets or exocrine pancreas.
15CP4	C	One section of pancreas was examined. Islets appeared slightly to moderately smaller than normal. Many islet cells were smaller than normal with reduced and minimally vacuolated cytoplasm. Rare insulin positive cells were present in the islets and in the exocrine pancreas.
15FP1	D	Three sections of pancreas were examined. Islets appeared moderately smaller than normal with reduced and minimally vacuolated cytoplasm. Rare insulin positive cells were present in the islets and in the exocrine pancreas.
15FP2	D	One section of pancreas was examined. Islets appeared moderately smaller than normal. Many islet cells were smaller than normal with reduced and minimally vacuolated cytoplasm. Rare insulin positive cells were present in the islets and in the exocrine pancreas.
15FP3	D	One section of pancreas was examined. Islets appeared moderately smaller than normal. Many islet cells were smaller than normal with reduced and minimally vacuolated cytoplasm. Rare insulin positive cells were present in the islets and in the exocrine pancreas.
13EP3	E	One section of pancreas was examined. Islets appeared normal in size to slightly smaller than normal. Many islet cells were smaller than normal with reduced cytoplasm. Only rare insulin positive cells were present in the islets and low numbers of scattered isolated insulin-positive cells were present between exocrine acini. The pancreas was otherwise within normal limits.
14HP21	E	Two sections of pancreas were examined. Islets appeared normal in size to slightly smaller than normal. Many islet cells were smaller than normal with reduced and minimally vacuolated cytoplasm. A few scattered insulin positive cells were present in the islets and in the exocrine pancreas.
14HP29	E	Two sections of pancreas were examined. Islets appeared normal in size to slightly smaller than normal. Many islet cells were smaller than normal with reduced and minimally vacuolated cytoplasm. A few scattered insulin positive cells were present in the islets and in the exocrine pancreas.
15FP13	E	Three sections of pancreas were examined. Islets appeared normal in size to slightly smaller than normal. Many islet cells were smaller than normal with reduced cytoplasm. Rare insulin positive cells were present in the interstitium of the exocrine pancreas.

Supplementary Table 7. Graft histopathology of Cohort B monkey # 15CP3.

Slide	*A*	*B*	*C*	*D*	*E*	*F1*	*F2*
#1	0	0	0	0	0	0	0
#2	0	0	0	0	0	0	0
#3	0	0	0	0	2	0	0
#4	0	0	0	0	0	0	0
#5	0	0	0	1	3	0	0
#6	0	0	0	0	0	0	0
#7	0	0	6	1	1	0	0
#8	0	0	1	4	1	0	0
#9	0	0	0	0	0	0	0
#10	0	0	1	1	1	0	0
**Totals**	**0**	**0**	**8**	**7**	**8**	**0**	**0**

Key to Table
A = Normal islet
B = Mild lymphocytic infiltrate surrounding islet
C = Moderate lymphocytic infiltrates with infiltration
D = Marked infiltrates with extensive loss of islet cells
E = Obliteration of islet with lymphocytic ± other inflammatory cell infiltrates as noted
F1 = Partial islet cell loss with fibrosis; no/minimal inflammation
F2 = Complete islet cell loss with fibrosis; no/minimal inflammation

Supplementary Table 8. Differentially expressed genes in Tr1 cells sorted from Cohort B and C monkeys grouped based on Reactome analysis.

Signal Transduction		
	EDGE test: cohort B vs cohort C tagwise dispersions	
Feature ID	Fold change	P value
ABI2	191.78	1.89E-03
ACKR1	1136.81	3.96E-03
ARHGEF38	1008.09	2.19E-03
CCR5	20.49	4.87E-03
CNKSR2	356.54	7.04E-03
CTTN	87.42	2.73E-03
DISP2	183.45	2.61E-03
DLG3	450.87	2.29E-04
GAB1	72.27	5.04E-03
GFRA1	107.2	7.10E-03
HIST1H4L	488.58	5.07E-03
ITGB3	259.36	5.00E-03
LRP5	183.98	5.35E-03
MAPK7	31.08	5.49E-03
MCF2L	391.04	4.75E-04
MIS12	90.09	6.24E-03
NCBP2	17.43	6.87E-03
NCK1	30.04	5.07E-03
P2RY2	235.71	4.15E-03
PMEPA1	95.68	5.75E-03
PRKAG1	17.32	7.96E-03
RGS16	39.93	3.99E-03
RGS18	215.98	3.55E-03
RNF2	218.7	3.32E-03
SH2D2A	21.11	3.54E-03
SKA2	25.53	5.64E-03
TUBB2B	53.27	2.74E-03

Metabolism		
	EDGE test: cohort B vs cohort C tagwise dispersions	
Feature ID	Fold change	P value
ACBD4	56.83	9.42E-03
ACOT7	72.59	9.50E-03
ADI1	88.84	3.70E-03
ADO	35.39	1.81E-03
COQ2	88.53	6.22E-03
CYB5R3	17.44	2.21E-03
CYP2C8	356.35	9.30E-03
GDPD5	99.21	4.19E-03
GK	82.51	9.38E-03
ISCA1	47.9	3.13E-03
LIPT1	72.99	9.35E-03
MCEE	23.58	4.40E-03
MMADHC	35.8	6.00E-03
MTRR	13.47	8.76E-03
NDUFB1	29.03	8.62E-03
NDUFS4	27.47	8.19E-03
PFKP	16.23	4.22E-03
PHKA1	42.34	2.30E-03
PRKAG1	17.32	7.96E-03
RPL7	1100.39	1.29E-04
RPSA	28.9	5.69E-03
SGMS2	498.32	7.37E-03
SLC27A2	93.21	5.46E-03
SULT4A1	512.47	5.82E-03
UGCG	25.35	5.83E-03
UGT2A1	356.35	9.30E-03
ZDHHC21	90.5	6.61E-03

Immune Activation		
	EDGE test: cohort B vs cohort C tagwise dispersions	
Feature ID	Fold change	P value
ABI2	191.78	1.89E-03
ANAPC11	98.37	3.75E-03
BATF	76.83	8.13E-03
CCR5	20.49	4.87E-03
CD300E	92.87	4.38E-03
CYB5R3	17.44	2.21E-03
DSC3	63.55	2.04E-03
HMGB1	20.04	7.53E-03
IFNLR1	43.08	8.41E-03
ISG15	61.67	8.45E-03
MAPK7	31.08	5.49E-03
MBL2	1024.92	2.22E-03
NANOG	60.09	7.55E-03
NCK1	30.04	5.07E-03
POLR3K	72.95	9.27E-03
PROS1	187.13	4.81E-03
SH2D2A	21.11	3.54E-03
SLC27A2	93.21	5.46E-03
TOM1	31.54	1.41E-03
TRIM68	33.12	4.01E-03
TUBB2B	53.27	2.74E-03

Gene Expression		
	EDGE test: cohort B vs cohort C tagwise dispersions	
Feature ID	Fold change	P value
E2F8	173.1	4.67E-03
HIST1H4L	488.58	5.07E-03
MOBP	694.57	8.32E-03
MYBL2	20.93	7.45E-03
NCBP2	17.43	6.87E-03
PLAGL1	20.18	8.92E-03
POLR3K	72.95	9.27E-03
PRKAG1	17.32	7.96E-03
RNF2	218.7	3.32E-03
SNAPC5	82.01	7.02E-03
TTF1	15.69	6.13E-03
ZNF181	20	8.94E-03
ZNF253	71.19	9.18E-03
ZNF398	19.22	8.55E-03
ZNF426	69.53	3.97E-03
ZNF441	35.1	2.29E-03
ZNF684	70.74	9.26E-03
ZNF688	134.9	3.00E-03
ZNF75D	26.53	7.43E-03

Supplementary Table 9. Top Canonical pathways in Tr1 cells.

ERK5 Signaling	6.99E-04
2-oxobutanoate Degradation I	1.11E-03
Mitochondrial Dysfunction	2.46E-03
Superpathway of Methionine Degradation	7.14E-03
NRF2-mediated Oxidative Stress Response	1.77E-02

Supplementary Table 10. MHC typing of donor-recipient pairs by pyrosequencing in Cohort A.

Cohort A

Animal ID	*Mamu* A Haplotype 1	*Mamu* A Haplotype 2	*Mamu* B Haplotype 1	*Mamu* B Haplotype 2	*Mamu* DRB Haplotype 1	*Mamu* DRB Haplotype 2
17EP12 (R)	A004	A004	B017a	B012b	**DR03a'**	DR016
17EP6 (D)	A008	A011	B001a	B045a	DR01a	**DR03a**
17EP18 (R)	A008	A025	B017a	B008	DR03f	**DR03a**
17EP7 (D)	A001	A004	B043a	B028	DR06	**DR03a**
17EP3 (R)	A004	A008	B024a	B047a	**DR03a**	DR14a
17EP23 (D)	A023	A025	B001a	B008	DR11c	**DR03a**

Supplementary Table 11. MHC typing of donor-recipient pairs by pyrosequencing in Cohort

B. **Cohort B**

**Animal ID**	***Mamu* A Haplotype 1**	***Mamu* A Haplotype 2**	***Mamu* B Haplotype 1**	***Mamu* B Haplotype 2**	***Mamu* DRB Haplotype 1**	***Mamu* DRB Haplotype 2**
13EP12 (R)	A002a	A001	B012a	B001a	DR16	**DR03a**
13EP11 (D)	A004	A023	B047a	B043a	**DR03a**	DR06
13EP13 (D)	A019	A023	B015c	B043a	**DR03a**	DR06
13EP8 (R)	A002a	A025	B001c	B015b	DR15a	**DR04a**
13EP9 (D)	A004	A004	B012b	B012b	DR04a	**DR04a**
14HP31 (R)	A004	A008	B056b	B028	**DR15a**	DR09a
14HP30 (D)	A004	A224a	B001a	B015a	DR03f	DR04a
14HP23 (D)	A002a	A001X	B015a	B055	**DR15a**	DR10
14HP26 (R)	A028	A004	B055	B002	DR03f	**DR06**
14HP36 (D)	A002a	A023	B015a	B043a	DR15a	**DR06**
14HP34 (R)	A001	A006	B047a	B043a	DR04a	**DR03f**
14HP35 (D)	A002a	A002a	B012a	B001c	**DR03f**	DR15a
15CP3 (R)	A019	A008	B015c	B069b	**DR03a**	DR04a
15CP14 (D)	A001	A004	B001a	B048	**DR03a**	DR01a
15CP6 (R)	A004	A004	B012b	B002	**DR04a**	**DR06**
15FP7 (D)	A001	A008	B055	B069b	DR03g	**DR04a**
15FP5 (D)	A012	A006	B001a	B043a	**DR06**	DR03f

Supplementary Table 12. MHC typing of donor-recipient pairs by pyrosequencing in Cohort C.

Cohort C

**Animal ID**	***Mamu* A Haplotype 1**	***Mamu* A Haplotype 2**	***Mamu* B Haplotype 1**	***Mamu* B Haplotype 2**	***Mamu* DRB Haplotype 1**	***Mamu*DRB Haplotype 2**
13EP5 (R)	A004	A004	B002	B069a	DR06	**DR03a**
13EP7 (D)	A008	A001	B015b	B047a	DR11a	**DR03a**
14HP33 (R)	A023	A002a	B043a	B012a	**DR06**	DR03f
14HP19 (D)	A004	A004	B028	B002	DR14a	**DR06**
15CP1 (R)	A023	A004	B043b	B001a	DR10	**DR04a**
15CP9 (D)	A008	A008	B028	B069b	DR09a	**DR04a**
15CP4 (R)	A006	A008	B043a	B001a	DR03f	**DR04a**
15CP7 (D)	A002a	A004	B012a	B012b	DR16	**DR04a**
14HP24 (R)	A006	A003	B024a	B093	**DR10**	DR11b
15FP11 (D)	A004	A001	B017a	B047a	**DR10**	DR04a

Supplementary Table 13. MHC typing of donor-recipient pairs by pyrosequencing in Cohort D

Cohort D

Animal ID	*Mamu* A Haplotype 1	*Mamu* A Haplotype 2	*Mamu* B Haplotype 1	*Mamu* B Haplotype 2	*Mamu* DRB Haplotype 1	*Mamu* DRB Haplotype 2
15FP01 (R)	A023	A028	B001a	B001a	DR04a	DR03f
15CP11 (D)	A002a	A001	B015a	B055	DR15a	DR03g
15FP02 (R)	A004	A004	B012b	B048	DR04a	DR16
14HP27 (D)	A001	A224a	B001a	B001a	DR01c	DR15a
15FP03 (R)	A008	A008	B017a	B028	DR04a	DR09a
15CP12 (D)	A049	A006	B071	B001a	DR01a	DR05a

Supplementary Table 14. MHC typing of donor-recipient pairs by pyrosequencing in Cohort E

Cohort E

Animal ID	*Mamu* A Haplotype 1	*Mamu* A Haplotype 2	*Mamu* B Haplotype 1	*Mamu* B Haplotype 2	*Mamu* DRB Haplotype 1	*Mamu* DRB Haplotype 2
14HP21(R)	A019	A023	B015c	B043a	DR03a	DR06
14HP20(D)	A004	A001	B012b	B001a	DR04a	DR03a
14HP29(R)	A028	A023	B055	B055	DR03f	DR01c
14HP28(D)	A001	A002a	B055	B015a	DR03g	DR15a
15FP13(R)	A004	A019	B056b	B015c	DR15a	DR03a
15CP10(D)	A002a	A002a	B015a	B015a	DR15a	DR15a
13EP03(R)	A002a	A008	B015b	B028	DR04a	DR09a
13EP4(D)	A004	A006	B012b	B024a	DR04a	DR10

Supplementary Table 15. Characteristics of ADL products infused in Cohort A.

Cohort A

**TEST/CELL TYPE**	**Day -7 Vaccine**	**Day+1 SPLC-Derived Vaccine**	**Day+1 PBL-Derived Vaccine**
**CRITICAL RAW MATERIALS**			
Spleen Weight (g)	7.0 ± 1.0	NA	NA
% Total SPLC for Expansion	NA	10 ± 0	NA
Total PBL Draw Volume per Donor (mL)	NA	NA	432.3 ± 9.6
**PRODUCT CHARACTERIZATION**			
# of Cells ($x10^9$ kg^{-1} Recipient BW)	0.149 ± 0.048	0.130±.017	0.113 ±.006
Total # of Cells Per Infusion ($x10^9$ kg^{-1} Recipient BW)	0.149 ±0.048	0.243 ± 0.023	
% Viable Cells♦	98 ± 2	96 ± 2	96 ± 2
Concentration of Cells Infused ($x10^{6ml-1}$)	18.6 ± 0.6	19.3 ± 1.2	19.6 ± 0.6
% Apoptotic Cells#	96 ± 2	95 ± 2	97 ± 3
% Necrotic Cells*	3 ± 2	5 ± 2	3 ± 2
% CD20+ Cells	NA	94.0 ± 2.6	92.7 ± 1.2
Microaggregates ≤1 per 100 µL (% of Products)	100	100	100
Total Endotoxin per Infusion ≤1.0 EU kg^{-1} (% of Products)	67	100	
Sterility Negative @ 14days (% of Products)	100	100	100
**EX-VIVO EXPANSION**			
Fold-Expansion♣	NA	48.9 ± 9.9	28.8 ± 12.0

NA: Not Applicable. PBL: Peripheral Blood Leukocytes. SPLC: Splenocytes. BW: Body Weight.

♦ by Acridine Orange/Propidium Iodide fluorometric assay # %
Annexin V+ cells after 4hrs at 37°C

*% Propidium Iodide+ cells

♣ After 8-34 days in culture

Supplementary Table 16. Characteristics of ADL products infused in Cohort C

Cohort C

**TEST/CELL TYPE**	**Day -7 Vaccine**	**Day+1 SPLC-Derived Vaccine**	**Day+1PBL-Derived Vaccine**
**CRITICAL RAW MATERIALS**			
Spleen Weight (g)	8.3 ± 2.9	NA	NA
% Total SPLC for Expansion	NA	18 ± 9	NA
Total PBL Draw Volume per Donor (mL)	NA	NA	395 ±74.6
**PRODUCT CHARACTERIZATION**			
# of Cells ($x10^9$ kg^{-1} Recipient BW)	0.217 ± 0.066	0.151 ±.071	0.040 ±.019
Total # of Cells Per Infusion ($x10^9$ kg^{-1} Recipient BW)	0.217 ± 0.066	0.190 ± 0.082	
% Viable Cells♦	99 ± 1	99 ± 1	99 ± 1
Concentration of Cells Infused ($x10^6$ml-1)	14.5 ± 4.2	13.7 ± 5.3	4.4 ± 1.1
% Apoptotic Cells#	96 ± 4	97 ± 1	96 ± 4
% Necrotic Cells*	2 ± 1	2 ± 1	2 ± 1
% CD20+ Cells	NA	92.5 ± 6.6	91.1 ± 6.6
Microaggregates ≤1 per 100 µL (% of Products)	100	100	100
Total Endotoxin per Infusion ≤1.0 EU kg^{-1} (% of Products)	100	60	
Sterility Negative @ 14days (% of Products)	100	100	100
**EX-VIVO EXPANSION**			
Fold-Expansion♣	NA	12.0 ± 6.8	2.6 ± 1.1

NA: Not Applicable. PBL: Peripheral Blood Leukocytes. SPLC: Splenocytes. BW: Body Weight.
♦ by Acridine Orange/Propidium Iodide fluorometric assay # %
Annexin V+ cells after 4hrs at 37°C
*% Propidium Iodide+ cells
♣ After 8-34 days in culture

Supplementary Table 17. Characteristics of ADL products infused in Cohort D

Cohort D

TEST/CELL TYPE	Day -7 Vaccine	Day+1 SPLC-Derived Vaccine	Day+1 PBL-Derived Vaccine
**CRITICAL RAW MATERIALS**			
Spleen Weight (g)	7.3 ± 2.1	NA	NA
% Total SPLC for Expansion	NA	12 ± 7	NA
Total PBL Draw Volume per Donor (mL)	NA	NA	285.3 ± 12.8
**PRODUCT CHARACTERIZATION**			
# of Cells (x10^9 kg^{-1} Recipient BW)	0.25 ± 0	0.138 ±.027	0.112 ±.027
Total # of Cells Per Infusion (x10^9 kg^{-1} Recipient BW)	0.25 ± 0	0.25 ± 0	
% Viable Cells♦	98 ± 1	99 ± 1	98 ± 1
Concentration of Cells Infused (x$10^6$$ml^{-1}$)	18.9 ± 0.8	19.3 ± 1.2	19.6 ± 0.6
% Apoptotic Cells#	97 ± 2	98 ± 1	97 ± 1
% Necrotic Cells*	3 ± 2	2 ± 1	2 ± 1
% CD20+ Cells	NA	96.5 ± 1.4	95.8 ± 1.3
Microaggregates ≤1 per 100 µL (% of Products)	100	100	100
Total Endotoxin per Infusion ≤1.0 EU kg^{-1} (% of Products)	100	33	
Sterility Negative @ 14days (% of Products)	100	100	100
**EX-VIVO EXPANSION**			
Fold-Expansion♣	NA	20.9 ± 2.3	11.3 ± 4.5

NA: Not Applicable. PBL: Peripheral Blood Leukocytes. SPLC: Splenocytes. BW: Body Weight.
♦ by Acridine Orange/Propidium Iodide fluorometric assay # %
Annexin V+ cells after 4hrs at 37°C
*% Propidium Iodide+ cells
♣ After 8-34 days in culture

Supplementary Table 18. Characteristics of ADL products infused in Cohort E

Cohort E

TEST/CELL TYPE	Day -7 Vaccine	Day+1 SPLC-Derived Vaccine	Day+1 PBL-Derived Vaccine
**CRITICAL RAW MATERIALS**			
Spleen Weight (g)	6.6 ±0.7	NA	NA
% Total SPLC for Expansion	NA	15.6 ± 0.5	NA
Total PBL Draw Volume per Donor (mL)	NA	NA	280.3 ± 125.7
**PRODUCT CHARACTERIZATION**			
# of Cells (x10^9 kg^{-1} Recipient BW)	0.25 ± 0	0.089 ± 64	0.101 ± 019
Total # of Cells Per Infusion (x10^9 kg^{-1} Recipient BW)	0.25 ± 0	0.243 ± 0.023	
% Viable Cells♦	100 ± 0	99 ± 1	0.197 ± 0.04
Concentration of Cells Infused (x10^6 mL^{-1})	15.4 ± 2.2	9.7 ± 5.1	16.2 ± 2.6
% Apoptotic Cells#	98 ± 0	98 ± 1	98 ± 1
% Necrotic Cells*	2 ± 1	2 ± 0	2 ± 1
% CD20+ Cells	NA	92.2 ± 4.4	94.6 ± 1.0
Microaggregates ≤1 per 100 µL (% of Products)	100	100	100
Total Endotoxin per Infusion ≤1.0 EU kg^{-1} (% of Products)	67	33	
Sterility Negative @ 14days (% of Products)	100	100	100
**EX-VIVO EXPANSION**			
Fold-Expansion♣	NA	20.8 ± 12.9	12.9 ± 5.9

NA: Not Applicable. PBL: Peripheral Blood Leukocytes. SPLC: Splenocytes. BW: Body Weight.
♦ by Acridine Orange/Propidium Iodide fluorometric assay # %
Annexin V+ cells after 4hrs at 37°C
*% Propidium Iodide+ cells
♣ After 8-34 days in culture

Supplementary Table 19. Characteristics of islet products transplanted in Cohort B.

Cohort B

Recipient ID	Donor ID	Post Cobe IE (by DNA)	Post Culture IE (by DNA)	Days in Culture	%Post Culture Recovery (by DNA)	Post Culture Purity (%)	Post Culture FDA/PI	IE Transplanted (by DNA)	IE kg^{-1} Transplanted (by DNA)	Endo-toxin (EU kg^{-1})
13EP12	13EP11	45558	20,425	7	45	95	86.7	20,425	3,128	2.97
13EP8	13EP9	43971	18,721	7	43	95	87.5	18,721	3,635	4.6
13EP12	13EP13	80515	23,106	6	29	95	88	23,106	3,538	2.43
14HP31	14HP30	18943	11,446	8	60	95	83.8	11,446	2,112	< 0.46
14HP31	14HP23	22961	19,761	7	86	95	84.5	19,761	3,646	< 0.46
14HP26	14HP36	39560	29,241	8	74	95	85.1	28,741	5,322	< 0.93
14HP34	14HP35	44742	41,538	8	93	95	92.7	36,000	10,000	< 0.97
15CP3	15CP14	59700	41,785	7	70	95	91.6	41,185	6,336	< 1.0
15FP13	15CP10	57561	35,045	6	61	95	89.1	34,445	4,160	< 0.97
15CP6	15FP7	26635	31,863	7	120	95	84.7	31,263	3,493	< 0.45
15CP6	15FP5	37942	25,338	2	67	95	86.6	24,738	2,764	< 0.45
Avg		43,463	27,115	7	68	95	87	26,348	4,376	1.426
SD		17,035	9,280	2	24	0	3	8,424	2,095	1.28
Min		18,943	11,446	2	29	95	84	11,446	2,112	0.45
Max		80,515	41,785	8	120	95	93	41,185	10,000	4.6

Supplementary Table 20. Characteristics of islet products transplanted in Cohort C.

Cohort C

Recipient ID	Donor ID	Post Cobe IE (by DNA)	Post Culture IE (by DNA)	Days in Culture	%Post Culture Recovery (by DNA)	Post Culture Purity (%)	Post Culture FDA/PI	IE Transplanted (byDNA)	IEkg^{-1} Transplanted (by DNA)	Endo-toxin (EU kg^{-1})
13EP5	13EP7	56337	37,391	8	61		87.4	37,000	5,547	<0.97
14HP33	14HP19	35902	38,407	7	107	95	90.6	37,907	7,234	<0.95
14HP24	15FP11	21483	22,172	7	103	95	80.2	21,572	6,181	<0.86
15CP4	15CP7	55055	34,468	7	63	95		33,868	5,132	<0.98
15CP1	15CP9	96437	60,271	7	62	95	89.2	59,671	13,686	<0.92
Avg		53,043	38,542	7	79	95	87	38,004	7,556	0.93
SD		25,250	12,313	0	21	0	4	12,309	3,146	0.043
Min		21,483	22,172	7	61	95	80	21,572	5,132	0.86
Max		96,437	60,271	8	107	95	91	59,671	13,686	0.98

Supplementary Table 21. Characteristics of islet products transplanted in Cohort D.

Cohort D

Recipient ID	Donor ID	Post Cobe IE (by DNA)	Post Culture IE (by DNA)	Days in Culture	%Post Culture Recovery (by DNA)	Post Culture Purity (%)	Post Culture FDA/PI	IE Transplanted (by DNA)	IEkg^{-1} Transplanted (by DNA)	Endo-toxin (EU kg^{-1})
15FP1	15CP11	32089	13,799	7	43	95	87.8	13,699	2,998	<0.50
15FP2	14HP27	47481	33,127	7	70	95	93.8	32,527	6,149	<0.95
15FP3	15CP12	56709	53,772	7	95	95	91.9	52,672	9,241	<0.88
Avg		45,426	33,566	7	69	95	91	32,966	6,129	0.78
SD		10,156	16,322	0	21	0	3	15,914	2,549	0.19
Min		32,089	13,799	7	43	95	88	13,699	2,998	0.5
Max		56,709	53,772	7	95	95	94	52,672	9,241	0.95

Supplementary Table 22. Characteristics of islet products transplanted in Cohort E.

Cohort E

Recipient ID	Donor ID	Post Cobe IE (by DNA)	Post Culture IE (by DNA)	Days in Culture	%Post Culture Recovery (byDNA)	Post Culture Purity (%)	Post Culture FDA/PI	IE Transp lanted (by DNA)	IE kg^{-1} Tran splan ted (by DNA)	Endo-toxin (EU kg^{-1})
15FP13	15CP10	57561	35,045	6	61	95	89.1	34,445	4,160	<0.97
14HP21	14HP20	34815	25,869	8	74	95	82.9	25,869	4,530	<0.88
14HP29	14HP28	68269	54,446	8	80	85	92.8	45,779	12,273	<0.94
13EP3	13EP4	50885	36,262	8	71	95	89.8	35,500	8,728	2.98
Avg		51,542	40,158	8	77	90	88	35,824	8,402	0.91
SD		16,727	14,289	0	3	5	5	9,955	3,872	0.03
Min		34,815	25,869	8	74	85	83	25,869	4,530	0.88
Max		68,269	54,446	8	80	95	93	45,779	12,273	0.94

Supplementary Table 23. Antibody clones used in immune mechanistic studies.

Marker	Label	Clone	Source	Cat#
CD2	PE	RPA-210	BL	300208
CD3	PerCP	SP 34-2	BD	552851
CD3	PECy7	SP 34-2	BD	557749
CD3	APCCy7	SP 34-2	BD	557757
CD3	BV500	SP 34-2	BD	560770
CD4	FITC	L200	BD	550628
CD4	PerCP	L200	BD	550631
CD4	PECy5	L200	BD	552838
CD4	APC	L200	Tonbo	20-0049
CD4	AF700	L200	BD	560836
CD8	FITC	RPA-T8	Tonbo	35-0088
CD8	PECy7	RPA-T8	Tonbo	60-0088
CD10	PE	HI10a	BD	557143
CD11b	PECy5	ICRF44	BL	301308
CD14	PE	M5E2	BD	5571154
CD15	FITC	80H5	BC	IM1423U
CD19	APC	J3-119	BC	IM2470U
CD19	AF700	J3-119	BC	PN A78837
CD19	FITC	4G7	BD	347543
CD20	PECy5	2H7	BD	555624
CD20	APC	2H7	Tonbo	20-0209
CD20	BV510	2H7	BL	302340
CD21	APC	B-ly4	BD	559867
CD24	APCCy7	SN3	Ab cam	Ab197137
CD25	APC	M-A251	BD	561399
CD27	FITC	MT271	BD	555440
CD27	PerCP	MT271	BD	564642
CD28	BV510	CD28.2	BL	302936
CD38	PECy7	HIT2	BD	560677
CD38	AF700	HIT2	BD	560676
CD39	PE	BU61	Ancell	188-050
CD40	PE	5C3	BD	555589
CD40	PECy5	5C3	BD	555590
CD45	BV500	D058-1283	BD	561489
CD45 RA	PECy7	5H9	BD	561216

Marker	Label	Clone	Source	Cat#
CD49b	FITC	AK-7	BD	555498
CD49b	BV510	AK-7	BD	742645
CD73	FITC	AD2	BD	561254
CD95	PECy7	DX2	BD	561636
CD107a	AF647	H4A3	BL	328612
CD122	PE	Mik-B2	BD	557323
CD127	PECy7	HIL-2R-M21	BD	560822
CXCR5	FITC	710D82.1	NHPRR	
LAG-3	PE	Polyclonal	R&D	FAB2319p
LAG-3	PerCP	Polyclonal	R&D	FAB2319C
PD-1	AF700	EH12-2H7	BL	329952
HLA-DR	eF700	L243	Tonbo	80-9952
HLA-DR	AF700	L243	BL	307626
IgD	PE	Polyclonal	SB	2030-09
IgM	FITC	G20-127	BD	555782
Tbet	PECy7	eBio4B10	eBio	255825-82
FoxP3	APC	206D	BL	320114
FoxP3	PE	206D	BL	320108
IL-10	AF647	JES3-9D7	BL	501412
TGF-β	PECy7	1MAb11	BL	502930
IFN-γ	FITC	B27	BL	506504
α-Hu Pure IL-10 (blocking)		JES3-9D7	BL	501407
Violet Proliferation Dye 450			BD	562158
CFSE Dye Cell Trace			Invitrogen	C34554
Ghost Dye™ UV 450			Tonbo	13-0863

BC: Beckman Coulter
BD: Becton Dickinson
BL: Bio Legend
NHP RR: Nonhuman Primates Reagent Resource
eBio: eBioscience

Supplementary Table 24. Tetramers used in the immune monitoring studies.

Tetramer	Source Antigen	Sequence	Position
HLA-DRB1*14:01	*Mamu*-A4	APVALRNLRGYYNQS	98
HLA-DRB1*14:01	*Mamu*-A8	SLRYFYTAVSRPGRG	28
HLA-DRB1*14:01	*Mamu*-A8	TRIYKAATQNYREGL	88
HLA-DRB1*14:01	*Mamu*-A1	SMKYFYTSMSRPGRG	28
HLA-DRB1*14:01	*Mamu*-A1	WEPFSQSTIPMVGII	298
HLA-DRB1*14:01	*Mamu*-A2/49	SMRYFYTSMSRPGRW	28
HLA-DRB1*03:01	*Mamu*-A4	TQFVRFDSDAASQRM	55
HLA-DRB1*03:01	*Mamu*-A8	TQFVRFDSDAESPRE	55
HLA-DRB1*03:01	*Mamu*-A2	APVNLRNLRGYYNQS	98
HLA-DRB1*14:01	*Mamu*-A2	APVNLRNLRGYYNQS	98
HLA-DRB1*03:01	*Mamu*-DR3a	YVRFDSDVGEHRAVS	66
HLA-DRB1*14:01	*Mamu*-DR4	GAGLFIYFRNQKGPS	243
HLA-DRB1*14:01	*Mamu*-DR1a	GAGLFIYFRNQKGHT	243

Supplementary Table 25. Primers used for TCR sequencing.

Primer name	Function	Sequence
Hering004_SmartCDS Oligo	cDNA synthesis primer	GACCGGCAATCTCTTCCTGG TTTTTTTTTTTTTTTTTTTTTTTTTTTTTT VN
Hrg004_TempSwUID	Template switch primer for cDNA synthesis	TGGTACGGGAACAGCACATC DDDDDTGTDDDDDTGTDDDDDrGrGrG
Hrg004_tcrC001_Nex Fwd	Targeted amplification of TCR VDJ	TCGTCGGCAGCGTCAGATGTGTATAAGA GACAG NNNTCTCTGCTTCTGATGGCTCA
Hrg004_tempSwitchC ust_NexRev	Targeted amplification of TCR VDJ	GTCTCGTGGGCTCGGAGATGTGTATAAGA GACAG NN TGGTACGGGAACAGCACATC
Nextera Read 1 Indexing Primer	For sample multiplexing	AATGATACGGCGACCACCGAGATCTACA C [i5 index] TCGTCGGCAGCGTC
Nextera Read 2 Indexing Primer	For sample multiplexing	CAAGCAGAAGACGGCATACGAGAT [i7 index] GTCTCGTGGGCTCGG
