# Supplementary material for: Long-term tolerance of islet allografts in nonhuman primates induced by apoptotic donor leukocytes
Source: Nat Commun. 2019 Aug 2;10:3495. doi: 10.1038/s41467-019-11338-y (PMC6677762; doi:10.1038/s41467-019-11338-y)
Supplement: Supplementary file 3 — Reporting Summary [file 41467_2019_11338_MOESM3_ESM.pdf]

nature research

Corresponding author(s): *Double-blind peer review submissions: write DBPR and your manuscript number here instead of author names.*

Reporting Summary

Nature Research wishes to improve the reproducibility of the work that we publish. This form provides structure for consistency and transparency in reporting. For further information on Nature Research policies, see Authors & Referees and the Editorial Policy Checklist.

Statistical parameters

When statistical analyses are reported, confirm that the following items are present in the relevant location (e.g. figure legend, table legend, main text, or Methods section).

n/a	Confirmed	
☐	☒	The exact sample size (*n*) for each experimental group/condition, given as a discrete number and unit of measurement
☐	☒	An indication of whether measurements were taken from distinct samples or whether the same sample was measured repeatedly
☐	☒	The statistical test(s) used AND whether they are one- or two-sided<br>*Only common tests should be described solely by name; describe more complex techniques in the Methods section.*
☒	☐	A description of all covariates tested
☒	☐	A description of any assumptions or corrections, such as tests of normality and adjustment for multiple comparisons
☐	☒	A full description of the statistics including central tendency (e.g. means) or other basic estimates (e.g. regression coefficient) AND variation (e.g. standard deviation) or associated estimates of uncertainty (e.g. confidence intervals)
☒	☐	For null hypothesis testing, the test statistic (e.g. *F*, *t*, *r*) with confidence intervals, effect sizes, degrees of freedom and *P* value noted<br>*Give P values as exact values whenever suitable.*
☒	☐	For Bayesian analysis, information on the choice of priors and Markov chain Monte Carlo settings
☒	☐	For hierarchical and complex designs, identification of the appropriate level for tests and full reporting of outcomes
☒	☐	Estimates of effect sizes (e.g. Cohen's *d*, Pearson's *r*), indicating how they were calculated
☐	☒	Clearly defined error bars<br>*State explicitly what error bars represent (e.g. SD, SE, CI)*

Our web collection on statistics for biologists may be useful.

Software and code

Policy information about availability of computer code

Data collection	All details of data collection are described in the methods section.
Data analysis	Data were analyzed using Microsoft Excel 2016, GraphPad Prism version 7, FlowJo version 10.1, as detailed under experimental procedures. Software used : Differentially expressed genes were identified using the edgeR (negative binomial) feature in CLCGWB (Qiagen, Valencia, CA) and Ingenuity Pathway Analysis Software (Qiagen, Valencia, CA) for pathway identification.

For manuscripts utilizing custom algorithms or software that are central to the research but not yet described in published literature, software must be made available to editors/reviewers upon request. We strongly encourage code deposition in a community repository (e.g. GitHub). See the Nature Research guidelines for submitting code & software for further information.

Data

Policy information about availability of data

All manuscripts must include a data availability statement. This statement should provide the following information, where applicable:

- Accession codes, unique identifiers, or web links for publicly available datasets
- A list of figures that have associated raw data
- A description of any restrictions on data availability

The data that support the findings of this study are available from the corresponding author upon reasonable request. All sequence data described in the manuscript are deposited with the GEO database with Accession Nos. GSE132691 and GSE132496.

Field-specific reporting

Please select the best fit for your research. If you are not sure, read the appropriate sections before making your selection.

☒ Life sciences ☐ Behavioural & social sciences ☐ Ecological, evolutionary & environmental sciences

For a reference copy of the document with all sections, see nature.com/authors/policies/ReportingSummary-flat.pdf

Life sciences study design

All studies must disclose on these points even when the disclosure is negative.

Sample size

The exploratory mechanistic studies (Supplementary Table 1, Cohort A) were designed to evaluate very specific questions unique to a novel apoptotic donor leucocyte (ADL) negative vaccination product with the aim to develop sensitive and specific assessment tools capable of satisfying a crucial unmet need in the field (in both humans and nonhuman primates (NHPs)). Specifically to predict achievement of a tolerant state and monitor anti-donor immunity, it is generally accepted to use group sizes with a minimum of 2-3 animals for exploratory immune mechanistic studies, with ideally 3 animals to avoid even split categorical data. These exploratory studies in Cohort A were not designed to achieve statistical power and significance.

The subsequent study of therapeutic efficacy and safety sample size were selected to evaluate the combined effect of ADL infusions under the cover of short-term immunotherapy together with selected recipient characteristics (as defined in Supplementary Table 1) on graft survival time in the absence of maintenance immunosuppression as compared with controls. The primary outcome measure was duration of allograft survival posttransplant in the NHP alloislet transplant model. The estimate of the survival function (fraction of islet allograft survival) was calculated using the Kaplan-Meier estimator. The Kaplan-Meier estimate (product-limit formula) is ideal for estimating proportion even where subjects are not observed to fail (censoring) and when the sample is rather small. Our NHP studies are designed with high regard for ethical considerations, the least number of animals possible, though not so few as to fail to detect biologically important effects or to necessitate the repetition of experiments. We used a historical estimator to determine the number of animals predicted to show a significant difference in survival times. In the NHP allotransplant model we base our sample size calculations on islet allograft survival times recorded in previous similar studies in our laboratory and to date in tolerance modeling, the group proportion was equal to 0.76 with a (large) expected effect size (difference between survival curves) varying by at least 50% between the experimental Cohort C (with ADL infusions) and the control Cohorts B (no ADL infusions). Using this estimate, we determined the smallest numbers needed using the Log-rank test of survival. To have at least 80% power to detect a significant result using a one sided test at the $\alpha=0.1$ level, allograft survival would need to be compared between groups containing ≥ 3 recipients.

Enrollment was increased in the control Cohort B and deliberately matched Cohort C (both MHC class I-disparate and 1 MHC class II DRB allele-matched donor-recipient NHP pairs approximating the intended target clinical populations) to determine statistical non-inferiority. Presuming up to 60% of recipients in the control Cohort B had the potential to meet the primary endpoint, with a specified non-inferiority margin of -20% and a one-sided alpha level of 0.05, a cohort size of 8 was planned in Cohorts B and C for an 80% power to determine statistical non-inferiority with the possibility of dropout in each Cohort, either a priori because of not meeting recipient characteristics (e.g., donor specific antibodies identified) or because of unrelated morbidity during the course of follow-up (not observed). The non-inferiority margin was selected based on the minimum clinically relevant difference, which in evaluations of immune tolerance appreciates the complexity in achieving and maintaining tolerance, resulting in a proportionally lower sample size.

Data exclusions

No animals were excluded from this analysis, complete details of all recipients are presented.

Replication

This study was performed as a single-center trial. Graft survival was scored at the time of euthanasia based on histology. Liver specimens were obtained from 10 different anatomical areas in each recipient. Sections from each of the 10 blocks were stained with hematoxylin & eosin (H&E) or immunostained for insulin to score transplanted islets.

Randomization

Recipient animals had an equal probability of cohort assignment within the provision of predefined numbers in each group. The different cohorts were processed identically throughout the entire experiment under established standard operating procedures using good research practice.

Blinding

Personnel performing direct clinical care were not blinded to cohort assignment. Clinical pathology, endocrine assessments, and anatomic pathology were analyzed under blinded conditions.

Reporting for specific materials, systems and methods

Materials & experimental systems

n/a	Involved in the study
☐	☒ Unique biological materials
☐	☒ Antibodies
☒	☐ Eukaryotic cell lines
☒	☐ Palaeontology
☐	☒ Animals and other organisms
☒	☐ Human research participants

Methods

n/a	Involved in the study
☒	☐ ChIP-seq
☐	☒ Flow cytometry
☒	☐ MRI-based neuroimaging

Unique biological materials

Policy information about availability of materials

Obtaining unique materials	anti-CD40 mAb is a mouse/rhesus chimeric IgG4 clone 2C10R4 and can be obtained from NIH Nonhuman Primate Reagent Resource, Mass Biologicals, Boston, MA, USA.

Antibodies

Antibodies used	All antibody clones used in the study have been validated by manufacturers and details catalog number and vendor information is provided in the supplementary materials section.
Validation	Antibody clones were selected based on the recommendations made by the NIH Nonhuman primate reagent resource (https://www.nhpreagents.org).

Animals and other organisms

Policy information about studies involving animals; ARRIVE guidelines recommended for reporting animal research

Laboratory animals	The cohorts included purpose-bred monkey (Macaca mulatta) donors and recipients of Indian origin obtained from the National Institute of Health and Infectious Diseases colony at AlphaGenesis, Inc, Yemassee, SC. Demographics of the recipient monkeys are presented in Supplementary Table 1.
Wild animals	Study did not involve wild animals
Field-collected samples	Study did not involve sample collected from the field.

Flow Cytometry

Plots

Confirm that:

- ☒ The axis labels state the marker and fluorochrome used (e.g. CD4-FITC).
- ☒ The axis scales are clearly visible. Include numbers along axes only for bottom left plot of group (a 'group' is an analysis of identical markers).
- ☐ All plots are contour plots with outliers or pseudocolor plots.
- ☒ A numerical value for number of cells or percentage (with statistics) is provided.

Methodology

Sample preparation	Samples were prepared according to standard protocols as detailed under experimental procedures.
Instrument	BD FACS Canto II
Software	Acquired with FACSDIVA version 6.1.3 and analyzed with FlowJo version10.1. software (TreeStar).
Cell population abundance	A minimum of 200,000 events were acquired. Purity of magnetic beads sorted and FACS sorted populations were analyzed by flow cytometry and were routinely >95% in these studies.

Gating strategy	Details regarding the gating strategy for each of the subset studied is described in detail in the supplementary materials sections with representative figures.

☒ Tick this box to confirm that a figure exemplifying the gating strategy is provided in the Supplementary Information.
